# 6-Shogaol inhibits HSCs activation and liver fibrosis by regulating glycolytic reprogramming via targeting HIF-1α

**DOI:** 10.1186/s13020-026-01396-y

**Published:** 2026-05-27

**Authors:** Junfa Yang, Min Shu, Hui Fang, Fan Yang, Jiahe Li, Yuansong Sun, He Li, Tao Xu

**Affiliations:** 1 Department of Emergency Surgery, The Second Affiliated Hospital of Anhui Medical University, Hefei, 230601 China; 2 Inflammation and Immune Mediated Diseases Laboratory of Anhui Province, School of Pharmaceutical Sciences, Anhui Medical University, Hefei, 230032 China; 3 Tongling People’s Hospital, Tongling, 244000 China; 4 School of Basic and Science Medicine, Anhui Medical University, Hefei, 230032 China; 5 Department of Ophthalmology, The First Affiliated Hospital of Anhui Medical University, Hefei, 230022 China

**Keywords:** Liver fibrosis, 6-Shogaol, Network pharmacology, Hepatic stellate cells, Glycolysis

## Abstract

**Background:**

Liver fibrosis is a representative scarring response that can ultimately lead to liver cancer. However, relevant antifibrotic drugs for the effective treatment of liver fibrosis in humans have not yet been identified. 6-Shogaol is derived from natural products and exhibits multiple biological activities, including anti-inflammatory and antioxidant properties; however, its efficacy and potential mechanism of action against liver fibrosis remains unclear. This study aimed to examine the anti-fibrotic properties and potential mechanisms of action of 6-Shogaol.

**Methods:**

Two liver fibrosis mouse models (carbon tetrachloride (CCl_4_) and bile duct ligation (BDL)) were constructed to evaluate the anti-fibrotic properties of 6-Shogaol *in vivo*. Transforming growth factor-β1 (TGF-β1)-induced human hepatic stellate cells (HSCs) LX-2 cells were used as *in vitro* models. Network pharmacology analysis was introduced to explore the key targets of 6-Shogaol regarding the mechanisms on liver fibrosis. Molecular docking, molecular dynamics simulations, drug affinity reactivity target stability (DARTS) and isothermal titration calorimetry (ITC) were used to detect the affinity and binding between 6-Shogaol and its target. Additionally, we invested the mechanism of 6-Shogaol through RNA sequencing combined with Western blotting, oxygen consumption rate (OCR), extracellular acidification rate (ECAR), immunofluorescence co-localization, histopathology, immunohistochemical staining and RT-qPCR.

**Results:**

The results showed that 6-Shogaol remarkably alleviated CCl_4_- and BDL-induced liver fibrosis in mice, including observations of improved liver function, decreased activity of HSCs, and decreased extracellular matrix (ECM) deposition. In an *in vitro* model, 6-Shogaol suppressed TGF-β1-induced LX-2 activation. Mechanistically, RNA sequence analysis revealed that the effect of 6-Shogaol on liver fibrosis is linked to glycolytic reprogramming. 6-Shogaol suppressed HSCs glycolysis by decreasing glycolytic enzymes (HK2, PKM2, and GLUT1) and glycolytic metabolite levels (lactic acid and pyruvic acid). Furthermore, network pharmacology suggested HIF-1α as a potential 6-Shogaol target. Molecular docking, molecular dynamics simulations, DARTS, and ITC confirmed that 6-Shogaol could directly bind to HIF-1α. Interestingly, we demonstrated that HIF-1α knockout significantly inhibited HSCs glycolysis and activation, whereas the overexpression of HIF-1α increased HSCs glycolysis and activation. Moreover, specific knockout of HIF-1α did not enhance the suppressive effect of 6-Shogaol on HSCs activation or fibrosis-associated protein expression *in vivo*.

**Conclusion:**

These findings showed that 6-Shogaol ameliorated liver fibrosis by modulating the expression of HIF-1α associated with glycolysis reprogramming and validate 6-Shogaol as a promising therapeutic strategy for liver fibrosis.

**Graphical Abstract:**

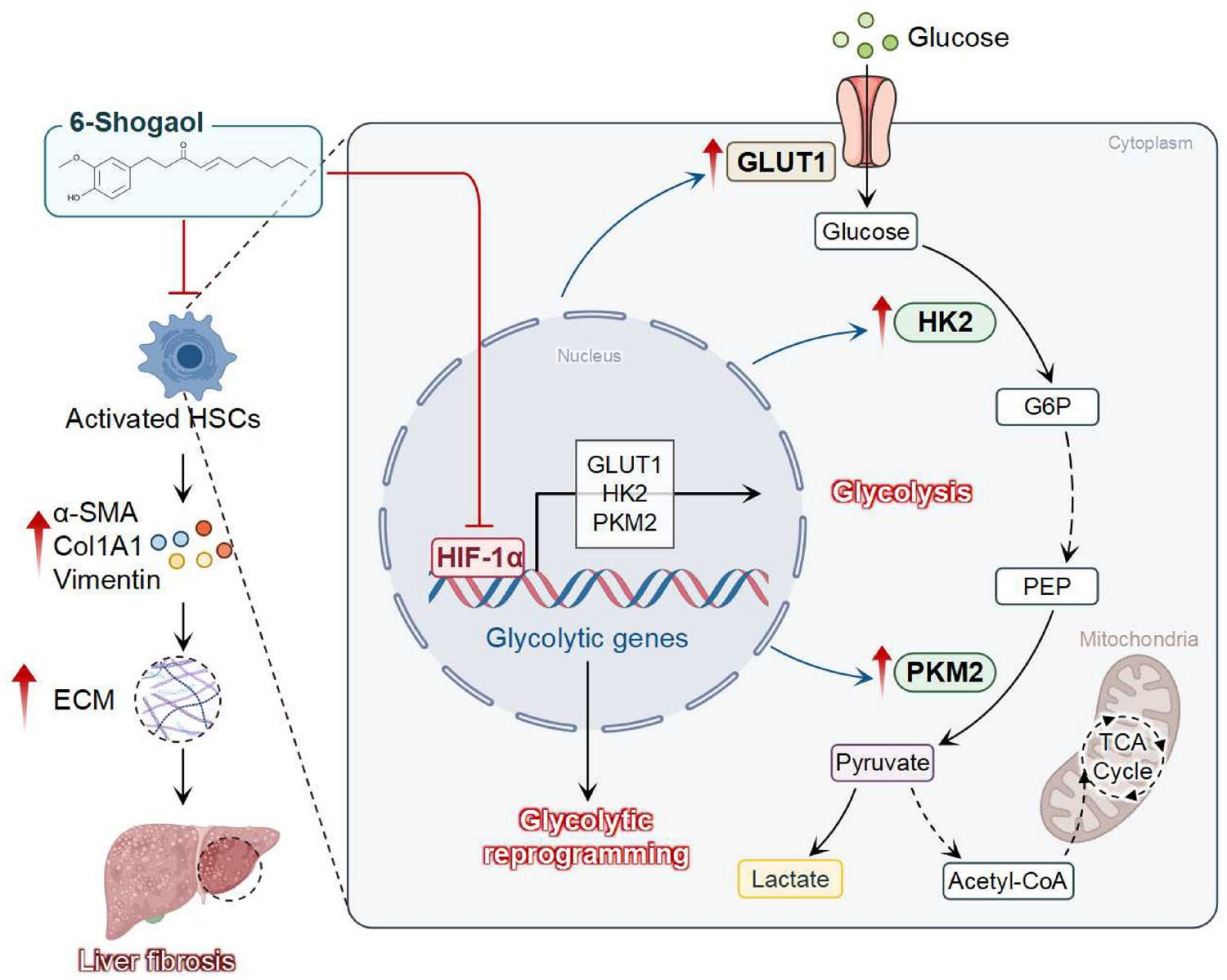

**Supplementary Information:**

The online version contains supplementary material available at 10.1186/s13020-026-01396-y.

## Introduction

Liver fibrosis is a central aspect of the progression of chronic liver disease and a major determinant of cirrhosis, liver carcinoma, and liver failure [1]. Therefore, the inhibition of fibrosis is crucial in preventing the progression of chronic liver diseases. Although several anti-fibrotic drugs have been researched, no approved therapies for liver fibrosis exist due to the complexity of its etiology, insufficient drug targeting and side effects [2–4]. Consequently, elucidating novel mechanisms of fibrosis and developing highly effective and low-toxicity antifibrotic agents is a current research priority.

Recent studies highlighted that metabolic dysfunction is a fascinating topic in the development of fibrosis across different organs [5, 6]. Among these, abnormal activation of glycolytic pathways is particularly striking. Activated pro-fibrotic cells, such as HSCs, are capable of both rapidly generating ATP to meet their bioenergetic requirements for proliferation (analogous to the Warburg effect mechanism) through enhanced glycolytic metabolism [7, 8], and providing essential precursors for the production of major components of the ECM [9, 10]. Recent studies have demonstrated that specific inhibition of key enzymes involved in glycolysis not only effectively dampens the activation phenotype of HSCs [11], but also significantly ameliorates liver fibrosis. This finding emphasizes the significant role of glycolysis in the pathogenesis of liver fibrosis. However, studies on altered metabolic profiles of HSCs in liver fibrosis and compounds targeting metabolic reprogramming remain scarce.

6-Shogaol, a principal component of dried ginger, possesses a range of pharmacological properties, such as antioxidant [12], anti-inflammatory [12, 13], and anti-cancer effects [14, 15]. Compared with fresh ginger extract, 6-Shogaol has superior biological activity and greater stability. Chen et al. have demonstrated that 6-Shogaol inhibited the growth and metastasis of breast and colon cancer and induced apoptosis through activation of peroxisome proliferator-activated receptor γ (PPARγ) [16]. The involvement of this nuclear receptor in the regulation of glycolipid metabolism suggests that it may influence the cellular energy metabolism network. Studies on liver diseases have reported that 6-Shogaol exhibits bidirectional regulation; on the one hand, it reduces hepatic lipid deposition by inhibiting lipid synthesis genes expression, and on the other hand, it optimizes glucose metabolism by promoting liver glycogen synthesis [17]. The establishment of this glucolipid metabolic balance is potentially linked to the regulation of the glycolytic pathway. Moreover, 6-Shogaol significantly attenuates hepatic inflammation and fibrosis by suppressing oxidative stress, reducing apoptosis and alleviating endoplasmic reticulum stress [18]. However, there are no reports on whether 6-Shogaol regulates glycolysis in HSCs to inhibit liver fibrosis.

Hypoxia-inducible factor-1 alpha (HIF-1α) is a member of the basic helix-loop-helix–PAS family of transcription factors [19]. It is a key mediator of cellular metabolism under hypoxic conditions, where it mediates reprogramming of cellular metabolism [20, 21]. HIF-1α promotes the progression of pulmonary fibrosis by mediating the regulation of lipid metabolic reprogramming [22, 23]. In addition, HIF-1α affects the occurrence and development of liver fibrosis by regulating oxidative stress, ferroptosis and other pathways [24–26]. Notably, HIF-1α transcriptionally regulates glucose metabolic reprogramming by mediating the expression of glycolytic genes, consisting of glucose transporters (GLUT1 and GLUT3), glycolytic enzymes (e.g. HK1, HK2, Eno1, and PKM2), and lactate dehydrogenase (LDHA) [27–30]. However, whether HIF-1α exerts its pro-fibrotic effects on liver fibrosis, specifically by modulating of glycolytic metabolism remains unclear.

In our current study, we preliminarily revealed that 6-Shogaol inhibited liver fibrosis and suppressed glycolytic reprogramming in vitro. In addition, we verified the ability of 6-Shogaol to decrease HIF-1α expression using *in vitro* experiments and investigated the effect of HIF-1α on therapeutic efficacy of 6-Shogaol using HIF-1α siRNA and pcDNA3.1-HIF-1α, both *in vivo* and* in vitro*. Overall, our findings provide insights into the regulation of glycolytic reprogramming as a novel therapeutic strategy for CCl_4_- and BDL-induced liver fibrosis.

## Materials & methods

### Network pharmacological analysis

#### Prediction of 6-Shogaol therapeutic targets

The chemical structure of 6-Shogaol (C_11_H_12_O_4_, CAS number: 555–66-8) was retrieved from PubChem (https://pubchem.ncbi.nlm.nih.gov) and drawn using the ChemDraw software (PerkinElmer, Waltham, MA, USA). Six publicly available databases were used, including BATMAN (http://bionet.ncpsb.org.cn/batman-tcm), CTD (http://ctdbase.org), GeneCards (https://www.genecards.org), HERB (http://47.92.70.12), TCMSP (https://www.tcmsp-e.com), and Switzerland (http://www.swisstargetprediction.ch) to predict the potential therapeutic targets of 6-Shogaol. “*Homo sapiens*” was selected as the species for all searches, and a probability threshold of > 0.1 was used where applicable. All targets obtained from the six databases were merged, and the reduplicated targets were deleted to obtain 6-shogaol therapeutic targets, which were standardized using the UniProt gene nomenclature (https://www.uniprot.org) for consistency in downstream analyses.

#### Identification of liver fibrosis-associated targets

The terms “liver fibrosis” and “hepatic fibrosis” were used as key words to search for liver fibrosis-associated targets in five databases: GeneCards (https://www.genecards.org), DisGeNET (https://www.disgenet.org), DrugBank (https://go.drugbank.com/), Online Mendelian Inheritance in Man (OMIM, https://omim.org/), and Therapeutic Target Database (https://db.idrblab.net/ttd). After retrieving the target genes from each database, appropriate filtering criteria were applied, based on the scoring system of each platform, to ensure their relevance to liver fibrosis. The resulting gene lists were merged. Duplicates and irrelevant genes were removed. Finally, a set of liver fibrosis-specific targets was compiled for subsequent analyses.

#### Construction of protein–protein interaction (PPI) network and identification of hub target genes

Overlapping genes between 6-Shogaol targets and liver fibrosis-associated genes were identified using a Venn diagram generated by Venny 2.1.0. These shared targets were used to construct a compound–disease–target interaction network using the Cytoscape software (version 3.10.3; https://cytoscape.org). In parallel, a protein–protein interaction (PPI) analysis was conducted using the STRING database (https://string-db.org), and the resulting network was visualized using Cytoscape. Node size and color intensity were used to represent the significance or centrality of each target gene [31].

Three topological analysis algorithms were applied using the CytoHubba plugin in Cytoscape to identify key regulatory genes (hub genes): (1) degree, which measures the number of direct interactions a node has with other nodes, (2) betweenness, which quantifies the number of shortest paths that pass through a node, indicating its control over communication in the network, and (3) maximal clique centrality (MCC), which evaluates the number and size of fully connected subgraphs (cliques) to which a node belongs, reflecting its role in densely connected network modules. The top-ranked genes from each algorithm were intersected using Venny 2.1.0 to identify robust hub targets with high topological significance.

#### GO and KEGG pathway enrichment analyses

Gene Ontology (GO) and Kyoto Encyclopedia of Genes and Genomes (KEGG) pathway enrichment analyses were performed using the Metascape database (https://metascape.org/) to investigate the biological functions and pathways associated with the shared targets of 6-Shogaol and liver fibrosis. The GO analysis covered three categories: biological processes (BP), cellular components (CC), and molecular functions (MF). The KEGG pathway analysis was used to identify signaling cascades relevant to fibrosis and inflammation. Furthermore, a comprehensive drug–disease–target–pathway network was constructed based on the enrichment results to elucidate the potential mechanisms of 6-Shogaol in liver fibrosis. The top 30 KEGG pathways ranked by statistical significance (–log_10_ *P*-value) were selected for network visualization. Corresponding hub genes enriched in these pathways were integrated into the final network using Cytoscape, facilitating the visualization of complex interactions among 6-Shogaol, liver fibrosis, core targets, and functional pathways.

### Animals and treatment

*C57BL/6 J* mice (6–8 weeks old) were provided by the Experimental Animal Center of Anhui Medical University. We generated a HIF-1α-HSC cKO mouse line (HIF-1α^Lrat−/−^), in which HIF-1α was specifically knocked down from HSCs. The conditional HIF-1α mice (HIF-1α^f/f^) were generated from Shanghai Model Organisms Center, Inc (Shanghai, China), HIF-1α^Lrat−/−^ mice were generated by crossing HIF-1α^f/f^ mice with Lrat-Cre transgenic mice, in which Cre recombinase is specifically expressed in HSCs. We used a dual-model approach to investigate liver fibrosis. As described previously, a subset of mice was intraperitoneally injected 15% CCl₄ (Sigma-Aldrich, St. Louis, MO, USA) oil twice weekly for 4 consecutive weeks to establish a chemically induced liver fibrosis model [32, 33]. A second model of liver fibrosis was established by using BDL. After anesthetizing the mice with 1.2–2.5 vol. % isoflurane, a 2-cm midline abdominal incision was created. The common bile duct was doubly ligated using 6–0 absorbable sutures and transected between the ligation sites in the model group. The common bile duct was isolated without ligation, and the incision was closed without performing BDL in the sham-operated group [34]. After initial model establishment, all model mice were gavaged daily with colchicine (MedChemExpress; Monmouth Junction, NJ, USA, #AMR69) and different concentrations of 6-Shogaol (MedChemExpress, #HY-N6966) daily. Previous studies have shown that the effective dose of 6-Shogaol was between 5 mg/kg and 100 mg/kg [18, 35–38]. In addition, before the formal study, we conducted preliminary experiments by using high and low doses in liver fibrosis models to screen out the optimal dose gradient that can produce a clear dose–response effect. Based on the safety data in the literature and the results of preliminary experiment, we selected gradients of 10, 20, and 40 mg/kg to establish a clear dose–response relationship and effectively cover the potential therapeutic range. The low dose (10 mg/kg) enabled us to determine the potential efficacy threshold, with the medium dose (20 mg/kg) aiming to achieve significant therapeutic effects. The higher dose (40 mg/kg) was used to explore the upper limit of efficacy within the safe range, ensuring that we could fully tap the potential of 6-Shogaol. Liver tissue and serum from the two distinct injury models were collected on days 42 and 21 after CCl₄ and BDL administration, respectively. All experimental procedures and animal care were conducted in accordance with the guidelines of the Laboratory Animal Research Committee of Anhui Medical University (approval no. LLSC20251114).

### Serum biochemical analysis

Blood was collected via retroorbital puncture of anesthetized mice, centrifuged, and the plasma was stored at -80 ℃. Commercial kits from Nanjing Jiancheng Bioengineering Institute (Nanjing, China) were used to assess liver injury by measuring the serum activities of alanine aminotransferase (ALT) and aspartate aminotransferase (AST), and levels of total bilirubin (TBIL), following the manufacturer’s instructions.

### Cell culture and drug treatment

Based on the findings of a previous study [32], a human HSC line (LX-2) was obtained from Shanghai Central Experiment Laboratory and cultured in Dulbecco’s modified Eagle’s medium (DMEM; Gibco, USA, #11,320–033). All culture media were complete, supplemented with 10% fetal bovine serum (FBS; Gibco, USA, #10,099–141), penicillin (100 μg/mL), and streptomycin (100 μg/mL) (Gibco, USA, #15,140–122). All cells were cultured under standard culture conditions (37℃, 5% CO2). To induce fibrosis, LX-2 cells were cultured until reaching 80%-85% confluence before passaging and were treated with or without 10 ng/mL transforming growth factor-beta (TGF-β1; 10 ng/mL, PeproTech; Rocky Hill, NJ, USA, #100–21) in serum-free medium. After 24 h, cells were exposed to final concentrations of 6.25, 12.5, and 25 μM of the test compound for an additional 24 h. 6-Shogaol was dissolved in dimethyl sulfoxide (DMSO; Aladdin, Shanghai, China) to prepare a stock solution of 20 mg/mL. The stock solution was diluted with DMSO to achieve final working concentrations of 0, 6.25, 12.5, and 25 μM. After treatment, the cells were collected for subsequent analyses.

### Histopathology and immunohistochemical (IHC) staining

All livers were fixed overnight at 4 °C in 4% (w/v) paraformaldehyde (Solarbio, Beijing, China) in 0.1 M sodium phosphate buffer (pH 7.4) and subsequently embedded in paraffin. The embedded tissues were sectioned at 4 μm thickness and stained with hematoxylin and eosin (H&E) for general histological evaluation. Masson’s trichrome and Sirius red staining were used to assess fibrosis. Liver tissue sections were deparaffinized in xylene, hydrated in 70%-100% (v/v) ethanol and phosphate-buffered saline (PBS) (pH 7.4), protected from endogenous peroxidase with 3% (w/v) H_2_O_2_, and finally encapsulated within 5% bovine serum albumin (BSA, Sigma-Aldrich, #A7906). Next, sections were incubated with primary antibodies overnight at 4℃. The primary antibodies used in our study for each marker are as follows: anti-α-smooth muscle actin (α-SMA) (1:100, Affinity, China), anti-collagen I (1:100, Wanlei, China), anti-HK2 (1:100, Proteintech, China), anti-pyruvate kinase M2 (PKM2) (1:100, Proteintech, China), anti-glucose transporter 1 (GLUT1) (1:100, Affinity, China), and anti- HIF-1α (1:100, Affinity, China). The slides were washed thrice with PBS, and then incubated with a secondary antibody at 37℃ for 30 min. Subsequently, diaminobenzidine (DAB; Aladdin, Shanghai, China) was used as the chromogen, and the sections were counterstained with hematoxylin. The sections were examined by using a laser scanning confocal microscope (Olympus; Tokyo, Japan), and representative images were acquired.

### Real-time quantitative polymerase chain reaction (RT-qPCR)

Total RNA was extracted from 50 mg of mouse liver tissues and LX-2 cells using the TRIzol reagent (Invitrogen, USA), following the manufacturer’s protocol. The samples were homogenized in the TRIzol reagent, and distinct RNA, DNA, and protein phases were obtained by adding chloroform and centrifuging. The RNA was precipitated from the aqueous phase using isopropanol, washed with 75% ethanol, and resuspended in RNase-free water. Complementary DNA (cDNA) synthesis was performed using the Evo M-MLV RT reagent kit (Accurate Biology; Wuhan, China) according to the manufacturer’s instructions. Real-time PCR was performed using the SYBR® Green Pro Taq HS qPCR kit (Accurate Biology) to estimate the expression of the identified genes. The relative gene expression was calculated using the 2^^−ΔΔCt^ method, with glyceraldehyde 3-phosphate dehydrogenase (GAPDH) as the internal reference gene. Each sample was analyzed in triplicate to ensure reproducibility. The primer sequences are listed in Table 1.

**Table 1:** Primer sequences for quantitative real-time reverse transcription polymerase chain reaction

Gene	Sequence
Collagen I	Forward PrimerGAGGGCCAAGACGAAGACATC Reverse PrimerCAGATCACGTCATCGCACAAC
Vimentin	Forward PrimerAGTCCACTGAGTACCGGAGAC Reverse PrimerCATTTCACGCATCTGGCGTTC
α-SMA	Forward PrimerCTATGAGGGCTATGCCTTGCC Reverse PrimerGCTCAGCAGTAGTAACGAAGGA
HK2	Forward PrimerGAGCCACCACTCACCCTACT Reverse PrimerCCAGGCATTCGGCAATGTG
PKM2	Forward PrimerATGTCGAAGCCCCATAGTGAA Reverse PrimerTGGGTGGTGAATCAATGTCCA
GLUT1	Forward Primer ATTGGCTCCGGTATCGTCAAC Reverse Primer GCTCAGATAGGACATCCAGGGTA
HIF-1α	Forward Primer TGGACTGGGTGCTTTGCTT Reverse Primer ATGATGGCGGTCTTGAACTG

### Western blotting analysis

Western blotting was performed as previously described [33, 39]. Total protein was extracted from the tissues and cells using radioimmunoprecipitation assay lysis (RIPA) buffer (Beyotime; Beijing, China) containing phenylmethanesulfonyl fluoride (PMSF, Beyotime), following the manufacturer’s protocol. The protein concentration was determined using the Pierce BCA Protein Assay Kit (Thermo Fisher Scientific; Rockford, IL, USA) according to the manufacturer’s instructions. The protein lysate was denatured, electrophoresed using 10% sodium dodecyl sulfate–polyacrylamide gel electrophoresis (SDS-PAGE), and transferred to PVDF membranes (Millipore; Billerica, MA, USA). After blocking with 5% skimmed milk for 1 h, the membranes were incubated overnight at 4℃ with primary antibodies, including anti-collagen I (1: 1000, Affinity, China), anti-α-SMA (1:1000, Affinity, China), anti-vimentin (1: 1000, Affinity, China), anti-β-actin (1: 1000, Affinity, China), anti-HK2 (1:1000, Proteintech, China), anti-PKM2 (1:1000, Proteintech, China), and anti-GLUT1 (1:1000, Proteintech, China), anti-lactate dehydrogenase A (LDHA, 1:1000, Proteintech, China), anti-HIF-1α (1: 1000, Affinity, China). The next day, the blots were incubated with goat anti-rabbit IgG secondary antibody (1: 1000; Affinity, China) and goat anti-mouse IgG secondary antibody (1:5000; Affinity) at room temperature for 1 h, and chemiluminescence was used to detect protein bands. Protein expression was normalized to that of β-actin, used as the internal control. Protein quantification was based on the gray values of the immunoblot bands, which were analyzed using the ImageJ software.

### RNA-seq

As mentioned above, total RNA was extracted from the liver tissues collected from CCl₄-induced mice and subsequently enriched and purified using magnetic beads. RNA-seq libraries were constructed according to standard protocols and sequenced on an Illumina NovaSeq 6000 platform (Personal Bio; Shanghai, China). Subsequently, differential expression gene (DEG) analysis was performed to compare the RNA expression profiles of the two groups. Transcripts with a false discovery rate (FDR) < 0.05 and fold change ≥ 2 were considered differentially expressed. The ClusterProfiler R package was used for GO enrichment analysis of DEGs.

### Assessment of lactic acid and pyruvic acid

Lactic acid and pyruvic acid levels in cell supernatants and mouse liver tissues were detected using lactic acid colorimetric analysis reagents (Nanjing Jiancheng, China, #A019-1–1) and pyruvate acid assay kit (Nanjing Jiancheng, China, #A081-1–1), respectively, according to the manufacturer's instructions. The samples were homogenized in ice-cold PBS, and the supernatant was collected after centrifugation. The assays were conducted by incubating the samples with the provided reagents, followed by measuring the absorbance at 570 nm for lactic acid and 540 nm for pyruvic acid using a microplate reader. The results were normalized to the total protein content, which was quantified using the BCA protein assay kit.

### Mitochondrial respiration and glycolysis assays

Mitochondrial respiration and glycolysis were evaluated using a Seahorse XF Glycolysis Rate Assay Kit (Agilent, USA). Cells were seeded in XF96 microplates at a density of 1 × 10^4^ cells per well and incubated overnight at 37 °C with 5% CO₂. On the second day, the medium was replaced with XF Assay Medium, and the basal oxygen consumption rate (OCR) was measured. Sequential injections of oligomycin (1 µM), FCCP (1 µM), and rotenone/antimycin A (1 µM) were administered to assess mitochondrial function. An extracellular acidification rate (ECAR) assay was performed to evaluate glycolytic function. Cells were incubated in glucose-free XF medium before sequential injections of glucose (10 mM), oligomycin (1 µM), and 2-deoxy-D-glucose (50 mM). Glucose addition stimulated glycolysis. Oligomycin was used to determine glycolytic capacity, and 2-deoxy-D-glucose (2-DG, Sigma-Aldrich, #D8375) served as a glycolysis inhibitor to confirm glycolysis dependence.

### Immunofluorescence (IF) assay

An immunofluorescence (IF) assay was conducted to assess the levels of LDHA and PKM2 in LX-2 cells, as previously described [39]. The cells were fixed with 4% paraformaldehyde, permeabilized with 0.3% Triton X-100, and blocked with 10% BSA. For single immunofluorescence, LX-2 cells were incubated overnight at 4 °C with anti-LDHA (1:300) and anti-PKM2 (1:300) antibodies. To examine the colocalization of HIF-1α and α-SMA, double immunofluorescence was performed by simultaneously incubating with anti-HIF-1α (1:300) and anti-α-SMA (1:300) antibodies. The secondary antibodies were Alexa Fluor 488 (green)-and Alexa Fluor 594 (red)-conjugated anti-rabbit antibodies. Nuclei were counterstained with 4, 6-diamidino-2-phenylindole (DAPI; Biyuntian, Shanghai, China). Images were captured using a fluorescence microscope.

### Molecular docking

Molecular docking was performed using the AutoDock Vina 1.1.2 software to dock 6-Shogaol (PubChem CID: 5,281,794) with the HIF-1α protein (UniProt ID: Q16665) [40]. Before docking, the proteins were prepared by removing water molecules and foreign ligands and adding hydrogen atoms using the PyMol 2.4 software. AutoDock Tools 1.5.6 was utilized to generate PDBQT files for docking simulations. The docking box was set with dimensions of 96 Å × 118 Å × 126 Å, a grid spacing of 1.00 Å, and coordinates of x:y:z:7.675:3.754:0.227. All other parameters were maintained at their default settings. The top 10 docking sites were selected, and the docking conformation with the lowest binding energy and the highest aggregation frequency was identified as the most favorable ligand–protein binding mode. Finally, the docking results were visualized using PyMol 2.4. The equation for converting the Vina fraction (ΔG) to Ki is expressed: ${\mathrm{Ki}}\, = \,{\mathrm{Kd}} = e^{{\frac{{\Delta {\mathrm{G}}}}{RT}}}$.

### Drug affinity reactivity target stability (DARTS) assay

The DARTS assay was conducted to detect the interaction between 6-Shogaol and HIF-1α in LX-2 cells. Briefly, LX-2 cells were lysed in HEPES buffer (containing 40 mM HEPES, pH 8.0, 120 mM NaCl, 10% glycerol, 0.5% Triton X-100, 10 mM β-glycerophosphate, 50 mM NaF, 0.2 mM Na_3_VO_4_, and protease inhibitors). The lysates were incubated with or without 6-Shogaol at room temperature for 1 h. After incubation, the samples were treated with pronase and incubated on ice for 30 min. The reaction was stopped by adding the SDS loading buffer, followed by boiling at 70 °C for 10 min. Subsequently, the protein samples were subjected to SDS–PAGE and western blotting using anti-HIF-1α and anti-β-actin antibodies. Protein stability was evaluated by comparing HIF-1α levels between treated and untreated groups.

### Small-interference RNA (siRNA)

The HIF-1α siRNA was designed and synthesized by Shanghai GenePharma Company (Shanghai, China), with the sequence GGGCCGUUCAAUUUAUGAATT (HIF-1α siRNA). Lipofectamine™ 3000 (Invitrogen, Waltham, MA, USA) was used according to the manufacturer’s protocol to transfect LX-2 cells with HIF-1α siRNA Silencer (HIF-1α siRNA) or nontargeting siRNA as a negative control (HIF-1α NC) [33].

### Plasmid transfection

The full-length HIF-1α cDNA was used as a template to construct a series of truncated HIF-1α plasmids and clone them into the pcDNA3.1 vector via restriction enzyme sites (XhoI and EcoRI). HIF-1α was transfected using Lipofectamine™ 3000 (Thermo Fisher Scientific) in accordance with the manufacturer’s protocols. The pcDNA3.1-HIF-1α expression plasmid or the empty pcDNA3.1 vector was used as a control for transfection.

### Isothermal titration calorimetry (ITC)

The binding models between 6-Shogaol and HIF-1α (Dingke Biotechnology Co., Ltd, China) were determined by isothermal titration calorimetry (ITC) using the Nano ITC instrument (Microcal Inc., Northampton, MA, USA). 6-Shogaol solutions (50 μL, 0.8 mM) in the syringe were titrated into the sample cell filled with HIF-1α (250 μL, 0.05 mM) with 250 s intervals between each injection; the temperature was maintained at 25 °C.

### Statistical analysis

All statistical analyses were performed using the SPSS software (version 21.0; IBM Corp. Armonk, NY, USA). The results are presented as the mean ± standard deviation (SD), and Pearson correlation analysis was performed to assess correlations. An unpaired *t*-test was applied for comparisons between two groups. One-way analysis of variance was used for comparisons among multiple groups, and Tukey's post hoc test was used for pairwise comparisons. Statistical significance was set at *P*-value < 0.05.

## Results

### Network pharmacology predicts the potential anti-fibrotic targets and mechanisms of 6-Shogaol

**Fig. 1:**
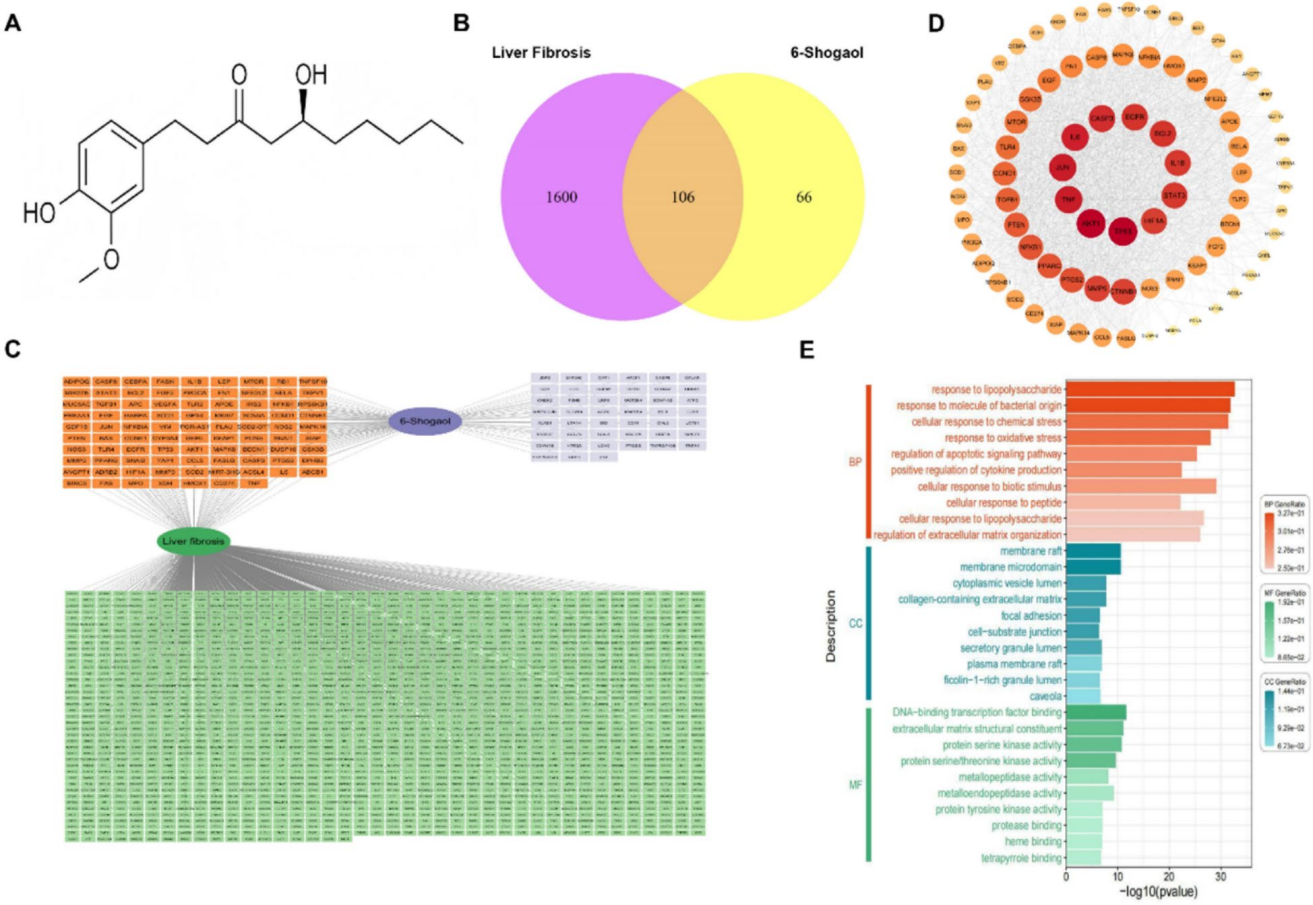
Network pharmacology analysis uncovers key targets and pathways of 6-Shogaol in liver fibrosis. **A** The chemical structure of 6-Shogaol. **B** Venn diagram comparing drug-related targets (purple) and disease-related targets (yellow), identifying 88 overlapping targets critical for liver fibrosis treatment. **C** 6-Shogaol-liver fibrosis- target network. **D** PPI network of the 88 overlapping target genes of 6-Shogaol and liver fibrosis. The larger size and darker color of a node indicate a higher degree of the target in the network. **E** GO analysis

Network pharmacology analysis was performed to explore the potential pharmacological mechanisms of 6-Shogaol in liver fibrosis. The chemical structure of 6-Shogaol (C₁₁H₁₂O₄) was retrieved and visualized (Fig. 1A). According to the TCMSP database, 6-Shogaol possesses favorable pharmacokinetic properties with an oral bioavailability (OB) of 41.85% and a drug-likeness (DL) index of 0.18, both of which meet the commonly used screening thresholds (OB ≥ 20% and DL ≥ 0.14) for active compounds in network pharmacology studies[31]. We predicted 1,706 potential therapeutic targets for liver fibrosis using disease target databases, whereas 172 target proteins were identified as possible targets of 6-Shogaol based on drug target databases. Among these, 106 overlapping targets were identified as therapeutic targets of 6-Shogaol in liver fibrosis (Fig. 1B). Moreover, an integrated drug–disease–target network was constructed to illustrate the interaction between 6-Shogaol and liver fibrosis through shared targets (Fig. 1C). A PPI network analysis (Fig. 1D) was constructed based on these overlapping targets, and core genes, such as TP53, AKT1, TNF, JUN, IL6, and HIF1A, were identified using topological algorithms. Their critical positions within the network suggested that they may serve as key targets for mediating the therapeutic effects of 6-Shogaol in liver fibrosis (Table 2). Furthermore, GO enrichment and KEGG pathway analyses were performed to investigate the biological functions of these overlapping genes. The targets were significantly enriched in processes such as the response to lipopolysaccharides, oxidative stress, and regulation of apoptotic signaling, as well as in functional pathways related to membrane rafts, ECM components, and protein kinase activity (Fig. 1E).

**Table 2:** Top 20 targets ranked by degree method in PPI network

Number	Gene name	Protein name
1	TP53	Tumor Protein p53
2	AKT1	V-akt murine thymoma viral oncogene homolog 1
3	TNF	Tumor Necrosis Factor
4	JUN	Jun Proto-Oncogene
5	IL6	Interleukin 6
6	CASP3	Caspase-3
7	EGFR	Epidermal Growth Factor Receptor
8	BCL2	B-cell lymphoma 2
9	IL1B	Interleukin 1 Beta
10	STAT3	Signal Transducer and Activator of Transcription 3
11	HIF1A	Hypoxia-Inducible Factor 1-alpha
12	CTNNB1	Catenin Beta-1 (Beta-catenin)
13	MMP9	Matrix Metallopeptidase 9
14	PTGS2	Prostaglandin-Endoperoxide Synthase 2
15	PPARG	Peroxisome Proliferator-Activated Receptor Gamma
16	NFKB1	Nuclear Factor Kappa B Subunit 1
17	PTEN	Phosphatase and Tensin Homolog
18	TGFB1	Transforming Growth Factor Beta 1
19	CCND1	Cyclin D1
20	TLR4	Toll-Like Receptor 4

### 6-Shogaol attenuated liver fibrosis in CCl_4_- and BDL-induced mice model

**Fig. 2.**
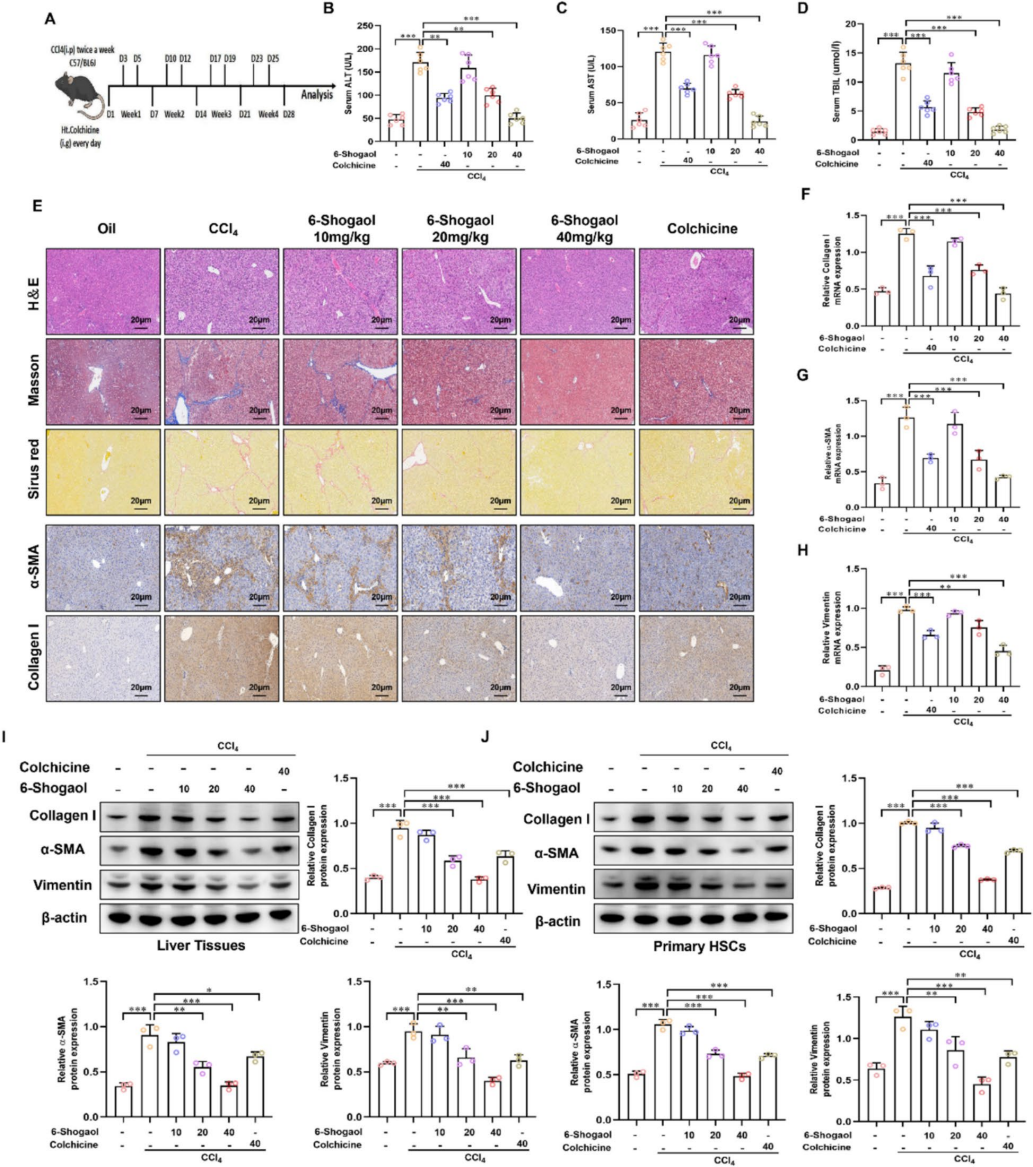
6-Shogaol attenuates liver fibrosis in the CCl_4_-induced mice model. **A** A flowchart describes the study design in CCl_4_-induced liver fibrosis. **B**, **C**, **D** The results of ALT, AST and TBIL in CCl_4_-induced mice. **E** The results of H&E staining, Masson’s trichrome, Sirius red, IHC-collagen I and IHC-α-SMA in liver fibrosis mice liver tissues. **F**, **G**, **H** The mRNA expression of collagen I, α-SMA and vimentin in mice liver fibrotic tissue. **I**, **J** The protein expression of collagen I, α-SMA and vimentin in mice liver fibrotic tissue and primary HSCs. *p < *0.05*, **p < 0.01, ***p < *0.01,* ns, not significant

**Fig. 3.**
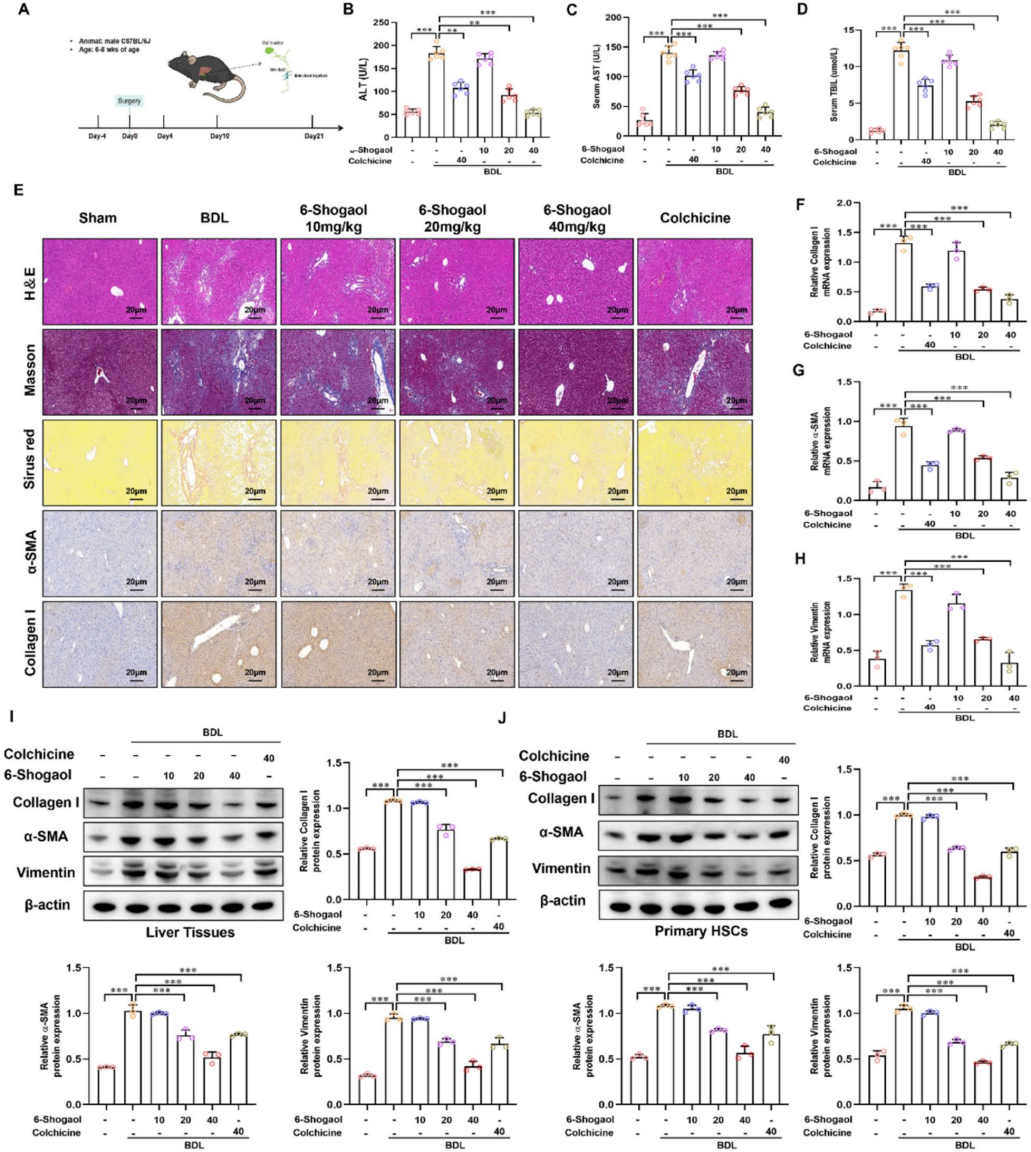
6-Shogaol attenuates liver fibrosis in the BDL-induced mice model. **A** A flowchart describes the study design in BDL -induced liver fibrosis. **B**, **C**, **D** The results of ALT, AST and TBIL in BDL -induced mice. **E** The results of H&E staining, Masson’s trichrome, Sirius red, IHC-collagen I and IHC-α-SMA in liver fibrosis mice liver tissues. **F**, **G**, **H** The mRNA expression of collagen I, α-SMA and vimentin in mice liver fibrotic tissue. **I**, **J** The protein expression of collagen I, α-SMA and vimentin in mice liver fibrotic tissue and primary HSCs. *p < *0.05*, **p < 0.01, ***p < *0.01,* ns, not significant

We established two distinct liver fibrosis models to observe the antifibrotic effect of 6-Shogaol: CCl_4_-induced liver fibrosis model and BDL-induced liver fibrosis model. Following fibrosis induction, mice were administered 6-Shogaol at doses of 10, 20, and 40 mg/kg via oral gavage for 4 weeks, with colchicine serving as a positive control (Fig. 2A, Fig. 3A). Serum biochemical analyses revealed that both CCl_4_ and BDL treatments greatly elevated ALT and AST activities and TBIL levels. Treatment with 6-Shogaol led to a dose-dependent normalization of these markers, suggesting improved liver function (Fig. 2B–D, Fig. 3B–D). Histopathological analysis using H&E staining revealed hepatocellular necrosis and inflammatory cell infiltration in fibrotic livers, whereas Masson’s trichrome and Sirius red staining indicated excessive collagen deposition and architectural distortion. Treatment with 6-Shogaol alleviated these pathological changes. Furthermore, IHC staining revealed markedly reduced expression of α-SMA and collagen I, key indicators of HSC activation and fibrosis progression (Fig. 2E, Fig. 3E). Molecular analyses by RT-qPCR and Western blotting further verified that 6-Shogaol reversed the significant up-regulation of mRNA and protein expression of collagen I, α-SMA and vimentin in a concentration-dependent manner in CCl_4_ and BDL groups (Fig. 2F–J, Fig. 3F–J). These findings consistently demonstrated that 6-Shogaol exhibited potent antifibrotic effects in vivo, indicating that despite differences in etiology, 6-Shogaol effectively mitigates liver fibrosis in both CCl_4_- and BDL-induced models, demonstrating its broad therapeutic potential.

### 6-Shogaol suppressed glycolytic reprogramming in fibrotic liver tissues

**Fig. 4.**
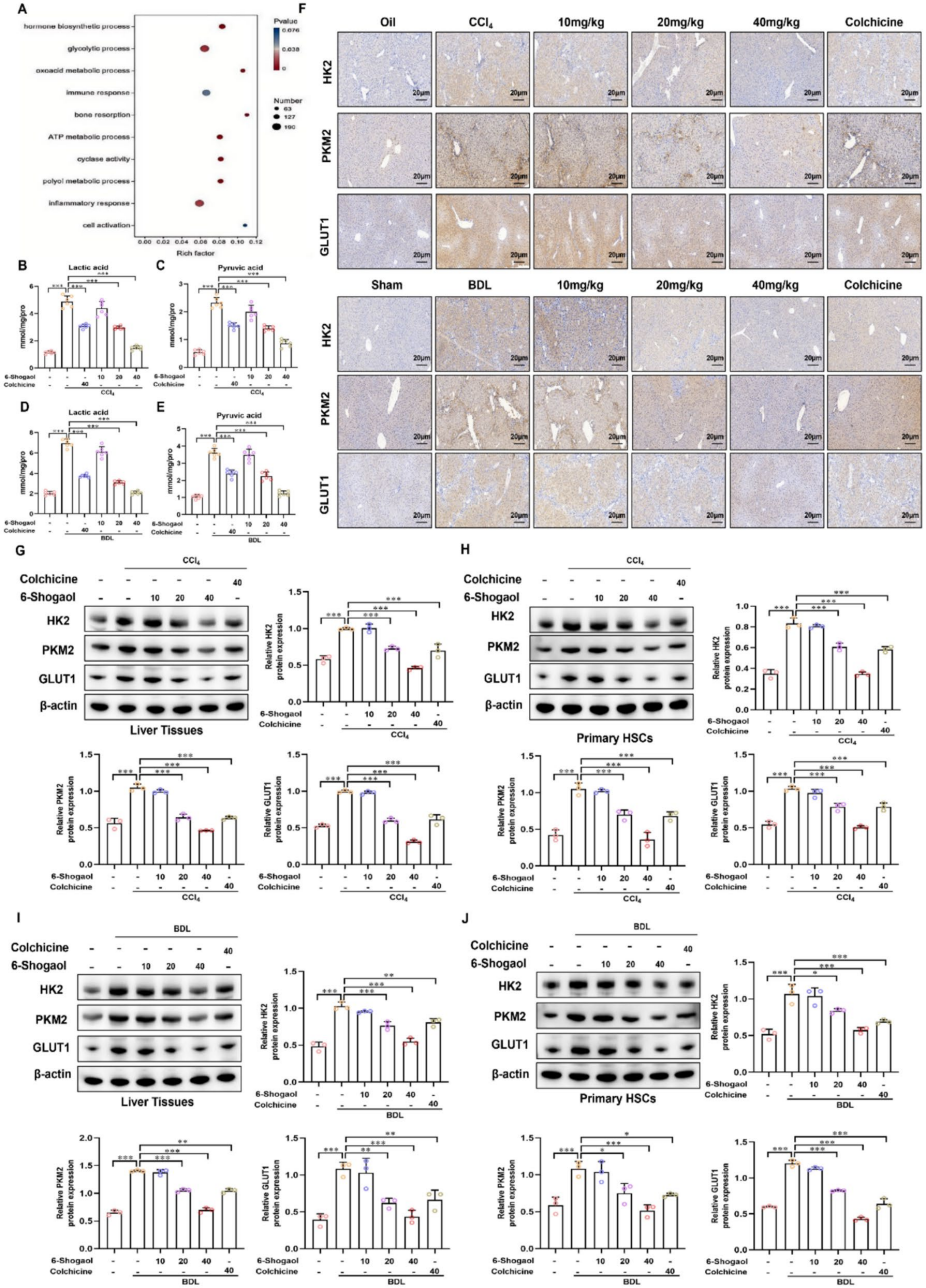
6-Shogaol suppresses glycolytic reprogramming in fibrotic liver tissue. **A** Gene Ontology (GO) was used for pathway enrichment analysis of differentially expressed genes in mouse liver fibrotic tissue. **B**, **C, D, E** Lactic acid and pyruvate acid content in mouse liver fibrotic tissue. **F** Results of IHC-HK2, IHC-PKM2, and IHC-GLUT1 in liver tissues of mice with liver fibrosis. **G**, **H**, **I**, **J** Expression of HK2, PKM2, and GLUT1 in the liver tissues of mice with liver fibrosis and primary HSCs. *p < *0.05*, **pp < 0.01, ***p < *0.01,* ns, not significant

Transcriptomic profiling of fibrotic liver tissues was performed to investigate the molecular mechanisms underlying the antifibrotic effects of 6-Shogaol. RNA-seq and subsequent GO enrichment analysis identified glycolytic reprogramming as one of the most significantly altered pathways following treatment with 6-Shogaol (Fig. 4A). Quantitative biochemical assays confirmed elevated levels of glycolytic metabolites such as lactate and pyruvate in fibrotic livers, which were strongly decreased following treatment with 6-Shogaol (Fig. 4B–E). In addition, IHC staining similarly verified the modulation of glycolytic metabolic by 6-Shogaol intervention, revealing the prevalence of glycolytic pathway activation during the course of liver fibrosis and its association with different etiologies (Fig. 4F). Similarly, we observed significant upregulation of HK2, PKM2 and GLUT1, which are glycolytic rate-determining enzymes, in CCl_4_-induced and BDL-induced mice by using Western blotting (Fig. 4G–J). Notably, the administration of 6-Shogaol reduced the expression of these enzymes in vivo in a dose-dependent manner. Taken together, these data indicated that the progression of liver fibrosis is closely associated with enhanced glycolytic activity in liver tissues, and that 6-Shogaol inhibited the glycolytic enzymes and the progression of fibrosis.

### 6-Shogaol reduced glycolysis and HSCs activation in TGF-β1-stimulated LX-2 cells

**Fig. 5.**
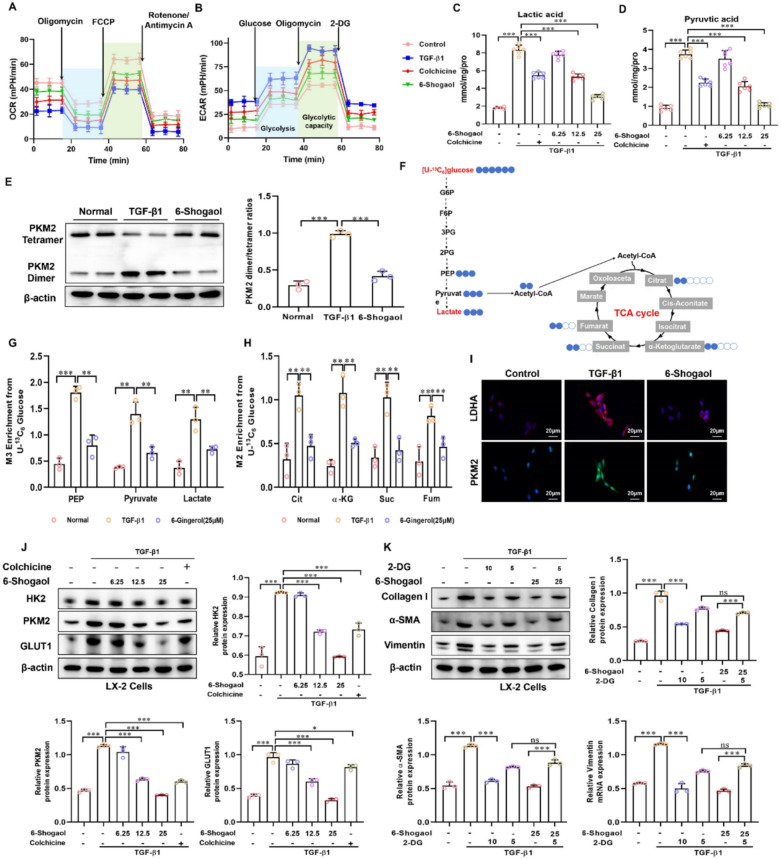
6-Shogaol reduces glycolysis and fibrotic activation in TGF-β1-stimulated LX-2 Cells. **A**, **B** Basal oxygen consumption rate (OCR) and extracellular acidification rate (ECAR) assessed by cellular energy metabolism experiments highlight metabolic alterations in the LX-2 cell. **C**, **D** Lactic acid and pyruvate acid content in LX-2 cells. **E** Detection of dimeric and tetrameric PKM2 protein levels in LX-2 cells. **F** Flux map and ^13^C-labeled glucose tracing of glycolysis and the TCA cycle. **G**, **H** Quantification of isotopes derived from ^13^C_6_-labeled glucose intermediates during glycolysis and the TCA cycle was performed using mass spectrometry. M + 3 indicates three carbon-labeled PEP, pyruvate, and lactate; M + 2 indicates two carbon-labeled citrate, α-ketoglutarate, succinate, fumarate, and malate. **I** Immunofluorescence staining was used to detect PKM2 and LDHA expression in LX-2 cells. **J**, **K** The protein expression of HK2, PKM2, GLUT1, collagen I, α-SMA, and vimentin in the LX-2 cell. *p and *#p* < *0.05*, **p and *##p* < 0.01, ***p and *###p* < *0.01*

To validate these findings in vitro, LX-2 cells were stimulated with TGF-β1 to induce fibrosis. Cell viability was detected using CCK8 kits in LX-2 cells treated with 6-Shogaol. The result showed that the cell viability was about 99.98%, 57.83% and 41.12% after treatment with 0 μM, 3.125 μM, 6.25 μM, 12.5 μM, 25 μM and 50 μM 6-Shogaol, respectively. Therefore, we selected 6.25 μg/mL, 12.5 μg/mL, and 25 μg/mL 6-Shogaol as optimal concentrations having the minimum cell toxicity (Supplementary Fig. S1A). Upon TGF-β1 stimulation, LX-2 cells exhibited increased ECAR and reduced OCR as measured by using Seahorse XF metabolic flux analysis, confirming a shift toward aerobic glycolysis. Treatment with 6-Shogaol effectively reversed these metabolic changes (Fig. 5A and B). In agreement with the metabolic changes, the levels of lactic acid and pyruvic acid in the cell culture medium were decreased following treatment with 6-Shogaol (Fig. 5C and D). Besides, we found that the tetrameric form of PKM2 was notably downregulated and replaced by dimeric PKM2 in LX-2 induced by TGF-β1. However, the PKM2 dimer/tetramer ratios were decreased treatment with 6-Shogaol (Fig. 5E). To confirm glycolysis and TCA cycle metabolic flux changes following treatment with 6-Shogaol were examined. [U–^13^C_6_]-glucose carbon tracing was performed to detect the labeled metabolites. The results demonstrated that [U–^13^C_6_]-PEP/pyruvate/lactate (M + 3) and [U–^13^C_6_]-Citrate/α-Ketoglutarate/Succinate/Fumarate/Malate (M + 2) were elevated in activated LX-2 cells. Interestingly, 6-Shogaol inhibited metabolite upregulation (Fig. 5F, G and F). IF and Western blotting revealed reduced expression of HK2, PKM2, and GLUT1, confirming that 6-Shogaol inhibited glycolytic enzyme expression in vitro (Fig. 5I and J). 2-DG, a glycolysis inhibitor, was used as a control in the presence or absence of 6-Shogaol to further illustrated the functional link between glycolysis and fibrosis. As depicted in Fig. 5K, treatment with 2-DG resulted in a strong antifibrotic effect in vitro, indicating that inhibition of glycolysis could inhibit liver fibrosis. In addition, 6-Shogaol (25 μM) effectively decreased the expression of collagen I, α-SMA and vimentin, and the combination of 2-DG (5 μM) blocked the antifibrotic effect of 6-Shogaol. These data suggested that the inhibition of glycolysis by 2-DG[41] obstructs the major antifibrotic pathway underlying the effects of 6-Shogaol. Thus, 6-Shogaol exerts its antifibrotic function by suppressing cellular glycolysis.

### 6-Shogaol directly targeted HIF-1α to inhibit liver fibrosis

**Fig. 6.**
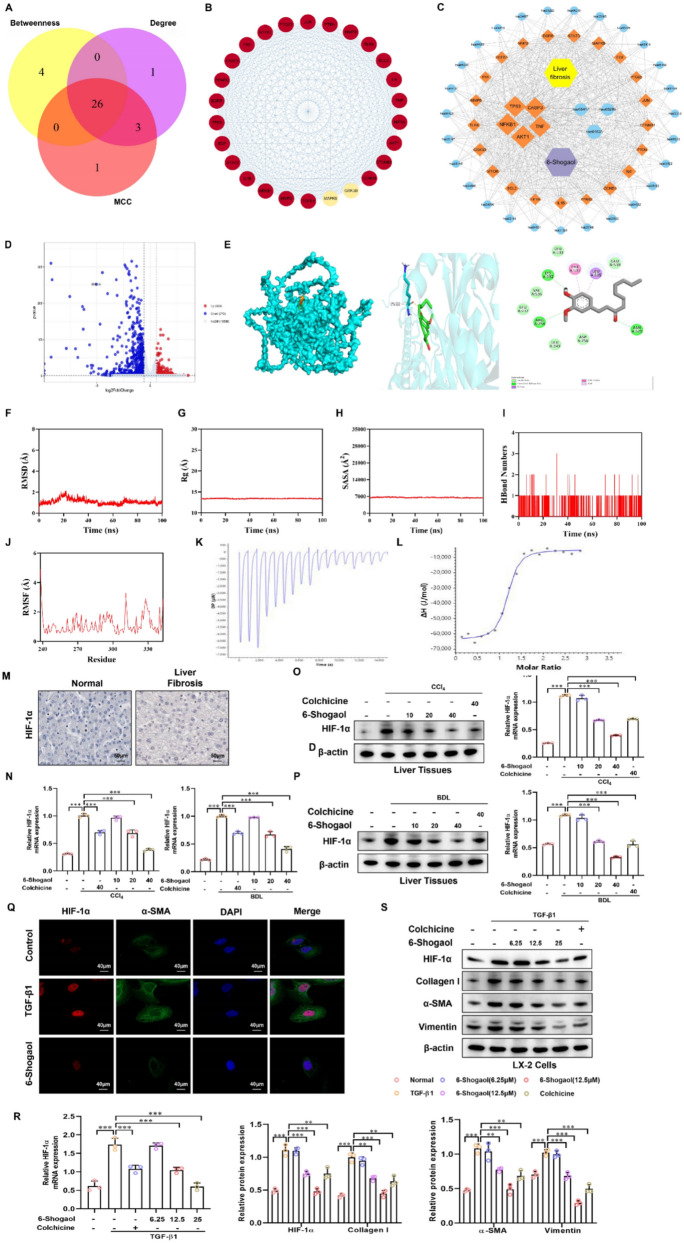
6-Shogaol directly targets and downregulats HIF-1α to inhibit fibrosis. **A**, **B** A total of 26 hub targets with the highest degree, betweenness, and maximal clique centrality were identified using a Venn diagram. **C** Integrated network of 6-Shogaol, liver fibrosis, hub genes, and KEGG pathways. **D** Volcano plot showing changes in protein levels between liver tissues of mice with liver fibrosis and liver tissues after treatment with 6-Shogaol. **E** Molecular docking studies revealed the direct interaction between 6-Shogaol and HIF-1α, highlighting the key residues involved in binding. **F** RMSD values of the four complexes. **G** Radius of gyration (Rg) values for the four complexes. **H** Solvent-accessible surface area (SASA) values of the four complexes. **I** Number of hydrogen bonds in the four complexes. **J** Values of the root-mean-square fluctuations. **K**, **L** The interaction between 6-Shogaol and HIF-1α was determined using the ITC analysis. **M** Immunohistochemical analysis of HIF-1α protein in the livers of patients with liver fibrosis by compared with healthy samples based on the data obtained from the Human Protein Atlas. **N** The mRNA expression of HIF-1α in mice liver fibrotic tissue. **O**, **P** The protein expression of HIF-1α in mice liver fibrotic tissue. **Q** Immunofluorescence staining was used to detect the results of HIF-1α in the LX-2 cells. **R** The mRNA expression of HIF-1α in the LX-2 cells. **S** The protein expression of HIF-1α, collagen I, α-SMA, and vimentin in the LX-2 cells. *p < *0.05*, **p < 0.01, ***p < *0.01*

Three network centrality algorithms (betweenness, degree, and MCC) were applied to the PPI network to further identify the potential therapeutic targets of 6-Shogaol in liver fibrosis, and 26 hub genes were identified based on their topological importance (Fig. 6A and B). Among them, HIF-1α was highlighted as a potential direct target based on its central role and relevance to metabolic regulation (Fig. 6C). Upon comparing the 6-Shogaol-treated group with the fibrosis group, the results of RNA-seq identified 1111 DEGs, comprising 339 upregulated genes and 772 downregulated genes. Interestingly, HIF-1α expression was greatly decreased in the 6-Shogaol-treated group, as illustrated in the volcano plot (Fig. 6D). Mechanistically, we applied the molecular docking technique to predict a favorable binding conformation between 6-Shogaol and HIF-1α and systematically evaluated it by using the AutoDock Vina software platform. The results revealed a stable binding between 6-Shogaol and HIF-1α using hydrogen bonding at the Lys-532 residue (Fig. 6E), suggesting a direct binding interaction. Molecular dynamics simulations were conducted to evaluate the interaction between 6-Shogaol and HIF-1α. The root mean square deviation (RMSD) values were used to assess whether the simulation systems reached a stable state. As shown in Fig. 6F, the RMSD values of the complexes stabilized at 2.2 Å. The radius of gyration (Rg) was analyzed to evaluate the compactness of the receptor-ligand binding. As depicted in Fig. 6G, the Rg values of the complexes remained stable throughout the simulation, reaching at 14 nm. The solvent-accessible surface area (SASA) was crucial parameter reflecting protein folding and stability. Moreover, the SASA values of the complexes demonstrated stability, reaching an average of 6900 Å^2^ (Fig. 6H). The number of hydrogen bonds reflected the strength of the protein ligand binding. The number of hydrogen bonds between the small molecule and the target protein ranged from 0 to 3. In most cases, the complex contained approximately one hydrogen bond, indicating that the protein ligand interacted well with the target protein via hydrogen bonding (Fig. 6I). Root mean square fuctuation (RMSF) indicated the flexibility of amino acid residues in proteins. As shown in Fig. 6J, the HIF-1α-Shogaol complex systems had relatively low RMSF values (mostly below 2 Å), making them less flexible and more stable. Subsequent analysis by using isothermal titration calorimetry (ITC) to measure heat changes during the binding of HIF-1α to 6-Shogaol validated that HIF-1α exhibited direct interaction with 6-Shogaol in terms of binding strength and thermodynamic parameters with a dissociation constant (KD) of 4.76E-05, suggesting that HIF-1α might function as a key target for 6-Shogaol. The binding stoichiometry (n) was found to be 1.1193, indicating that approximately one 6-Shogaol molecule binds to one HIF-1α protein molecule (Fig. 6K and L). DARTS assays [42] further confirmed enhanced resistance of HIF-1α to proteolysis upon treatment with 6-Shogaol (Supplementary Fig. S1B). In brief, the above results indicated that 6-Shogaol may form a direct binding interaction with HIF-1α. In addition, IHC showed markedly increased expression of HIF-1α in fibrotic liver tissues (Fig. 6M). Furthermore, RT-qPCR and Western blotting confirmed that HIF-1α expression was significantly upregulated in CCl_4_- and BDL-induced liver fibrosis mice compared to the oil and sham group. Consistent with the previous experiment, 6-Shogaol inhibited HIF-1α expression in a dose-dependent manner in vivo (Fig. 6N–P). Double IF staining revealed that HIF-1α co-localized with α-SMA in the liver tissues (Supplementary Fig. S1C). We used an in vitro fibrosis model by treating TGF-β1 (10 ng/ml) in LX-2 cells for 24 h to further investigate the potential antifibrotic effects of 6-Shogaol. Colchicine has been used as a positive control drug both in vivo and in vitro [43]. RT-qPCR, Western blotting, and IF illustrated that HIF-1α expression was effectively suppressed by 6-Shogaol (Fig. 6Q–S). Collectively, HIF-1α was elevated in liver fibrosis and 6-Shogaol could target HIF-1α.

### HIF-1α promoted glycolysis and fibrogenesis in LX-2 Cells

**Fig. 7:**
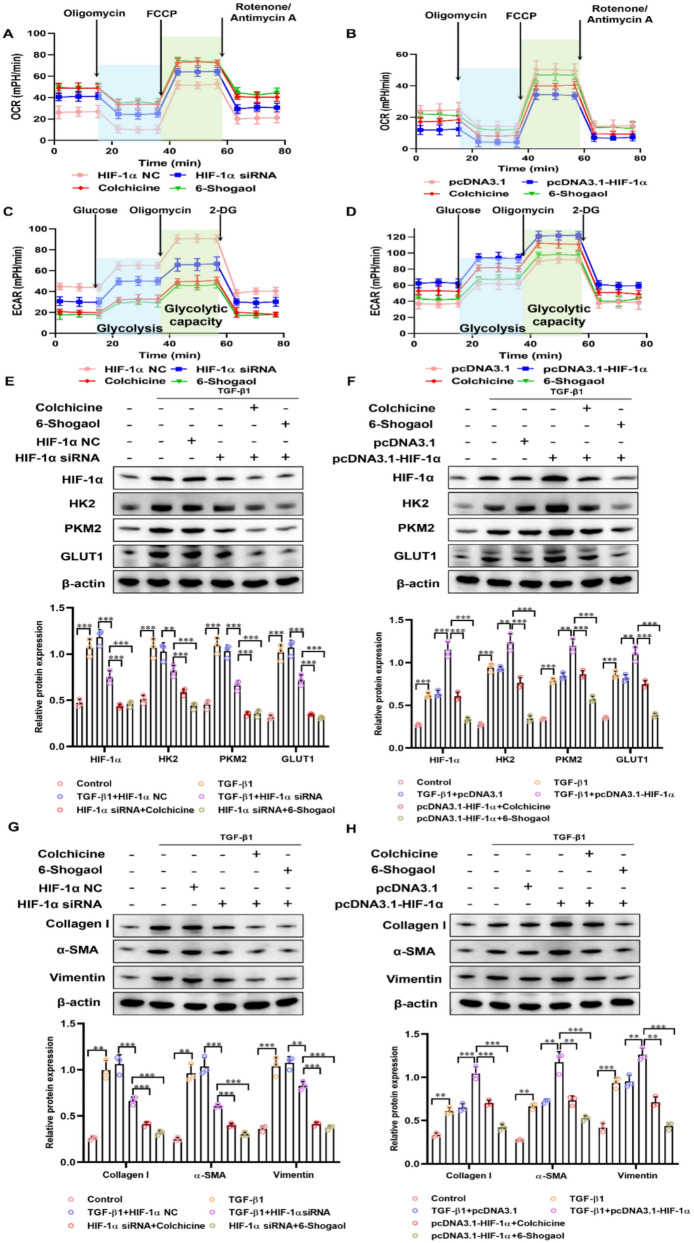
HIF-1α promotes liver fibrosis and glycolysis in the LX-2 cells. **A**, **B** Basal oxygen consumption rate (OCR) assessed by cellular energy metabolism experiments highlight metabolic alterations in LX-2 Cells treated with HIF-1α siRNA and pcDNA3.1- HIF-1α. **C**, **D** Basal extracellular acidification rate (ECAR) assessed by cellular energy metabolism experiments highlight metabolic alterations in LX-2 Cells treated with HIF-1α siRNA and pcDNA3.1- HIF-1α. **E**, **F, G, H** The protein expression of HIF-1α HK2, PKM2, GLUT1, collagen I, α-SMA and vimentin in LX-2 Cells treatment with HIF-1α siRNA and pcDNA3.1- HIF-1α. *p < *0.05*, **p < 0.01, ***p < *0.01,* ns, not significant

Next, LX-2 cell lines were transfected with HIF-1α siRNA and pcDNA3.1-HIF-1α to overexpress or silence HIF-1α and explored the functional role of HIF-1α in glycolytic metabolism and fibrogenesis. Seahorse analysis revealed that HIF-1α silencing significantly reduced both OCR and ECAR, indicating the suppression of mitochondrial respiration and glycolysis (Fig. 7A and C). In contrast, HIF-1α overexpression markedly increased OCR and ECAR, suggesting enhanced glycolytic metabolism (Fig. 7B and D). Treatment with colchicine or 6-Shogaol effectively counteracted the HIF-1α-induced increases in OCR and ECAR. These results were confirmed by Western blotting. In the context of TGF-β1 stimulation, HIF-1α knockdown significantly decreased levels of glycolysis-related proteins (HK2, PKM2, and GLUT1) (Fig. 7E) and fibrosis-related markers (collagen I, α-SMA, and vimentin) (Fig. 7G). Conversely, overexpression of HIF-1α significantly upregulated these protein levels (Fig. 7F and H), an effect which was notably reversed by colchicine and 6-Shogaol treatments. Collectively, all these findings emphasized the pivotal role of HIF-1α in modulating glycolytic pathways during fibrosis progression, and that 6-Shogaol inhibited glycolytic reprogramming by targeting HIF-1α to reverse liver fibrosis.

### HIF-1α knockout mitigates glycolytic reprogramming and liver fibrosis in vivo

**Fig. 8:**
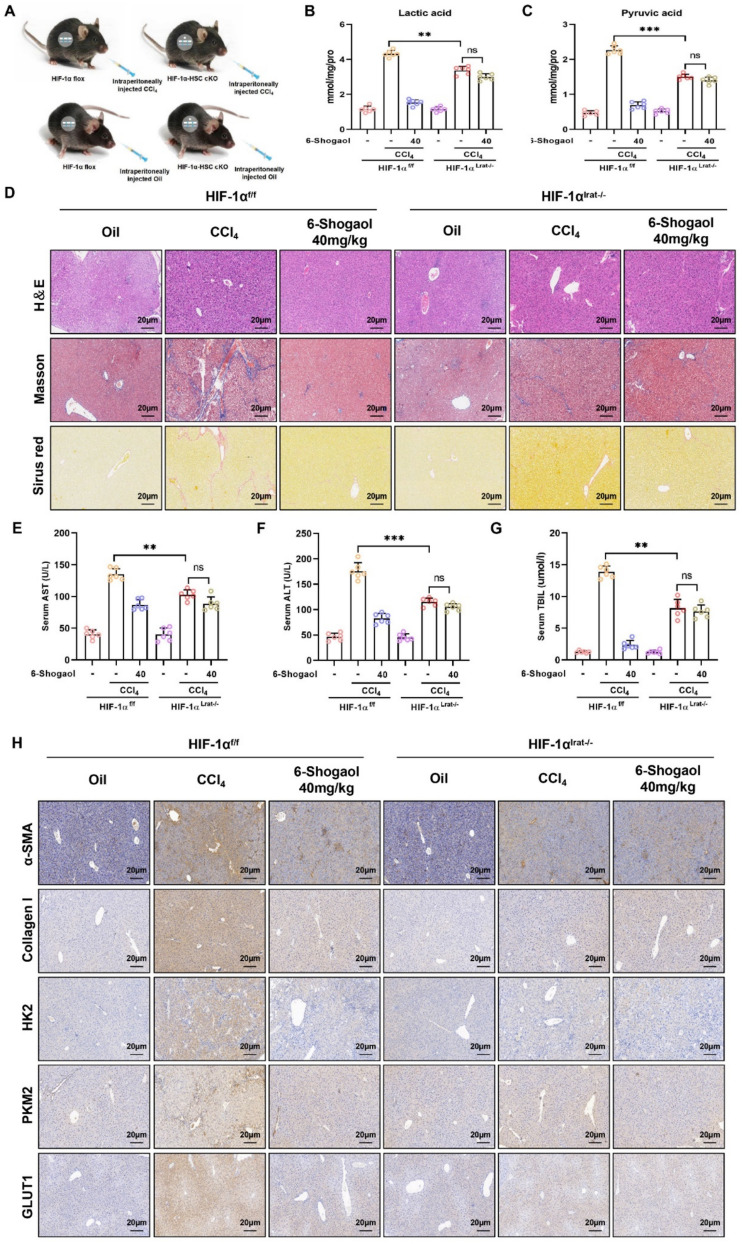
HIF-1α knockout mitigates liver fibrosis and glycolytic reprogramming in the CCl_4_-induced mice model. **A** Flowchart describes the study design in HIF-1α-cKO mice or vector mice followed with CCl_4_-induced liver fibrosis. **B**, **C** The contents of lactic acid and pyruvate acid in mice liver fibrotic tissue. **D** The results of H&E staining, Masson’s trichrome and Sirius red in liver fibrosis mice liver tissues. **E**, **F**, **G** The results of ALT, AST and TBIL in CCl_4_-induced mice. **H** The results of IHC-collagen I, IHC-α-SMA, IHC-HK2, IHC-PKM2 and IHC-GLUT1 in liver fibrosis mice liver tissues. *p < *0.05*, **p < 0.01, ***p < *0.01,* ns, not significant

**Fig. 9:**
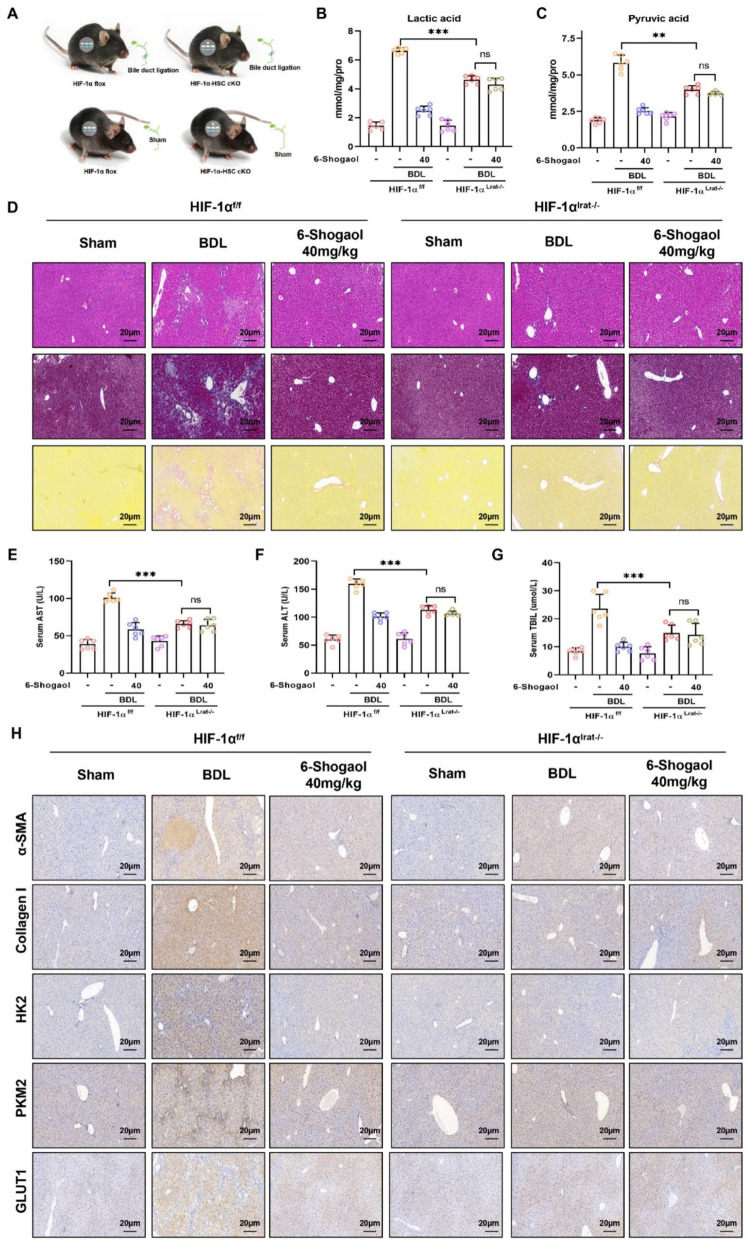
HIF-1α knockout mitigates liver fibrosis and glycolytic reprogramming in the BDL-induced mice model. **A** Flowchart describes the study design in HIF-1α-cKO mice or vector mice followed with BDL-induced liver fibrosis. **B**, **C** The contents of lactic acid and pyruvate acid in mice liver fibrotic tissue. **D** The results of H&E staining, Masson’s trichrome and Sirius Red in liver fibrosis mice liver tissues. **E**, **F**, **G** The results of ALT, AST and TBIL in CCl_4_-induced mice. **H** The results of IHC-collagen I, IHC-α-SMA, IHC-HK2, IHC-PKM2 and IHC-GLUT1 in liver fibrosis mice liver tissues. *p < *0.05*, **p < 0.01, ***p < *0.01,* ns, not significant

HIF-1α^lrat−/−^ mouse models were constructed and subjected to CCl_4_- and BDL-induced liver fibrosis, with or without 6-Shogaol treatment to confirm the metabolic regulatory mechanisms of HIF-1α in liver fibrosis, as illustrated in the flowchart (Fig. 8A and Fig. 9A). Baseline levels of lactic acid and pyruvic acid were significantly reduced compared to HIF-1α^f/f^ mice in the absence of HIF-1α, suggesting that HIF-1α knockdown suppressed glycolytic reprogramming and fibrogenesis. Additionally, metabolic analyses revealed that 6-Shogaol markedly decreased lactic acid and pyruvic acid levels elevated by CCl₄ or BDL in HIF-1α^f/f^ mice. However, this metabolic suppression effect of 6-Shogaol was abolished in HIF-1α^lrat−/−^ mice, indicating that the regulatory function of 6-Shogaol on glycolytic reprogramming was HIF-1α-dependent (Fig. 8B, C and Fig. 9B, C).

**Fig. 10:**
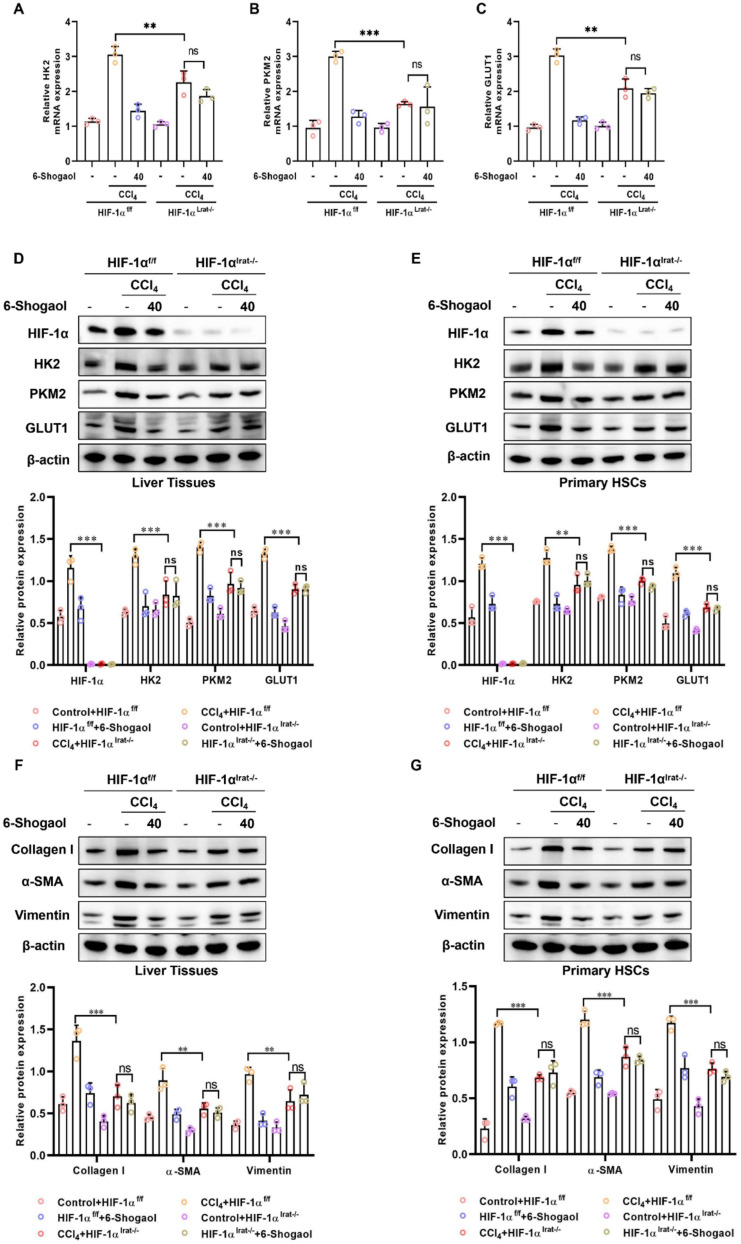
HIF-1α deletion attenuates the anti-glycolytic and anti-fibrotic effects of 6-Shogaol in CCl_4_-induced liver fibrosis and in primary HSCs. **A**, **B**, **C** The mRNA expression of HK2, PKM2 and GLUT1 in mice liver fibrosis tissues. **D**, **E** The protein expression of HIF-1α, HK2, PKM2 and GLUT1 in liver tissues and primary HSCs. **F**, **G** The protein level of collagen I, α-SMA, vimentin in liver tissues and primary HSCs. *p < *0.05*, **p < 0.01, ***p < *0.01,* ns, not significant

**Fig. 11:**
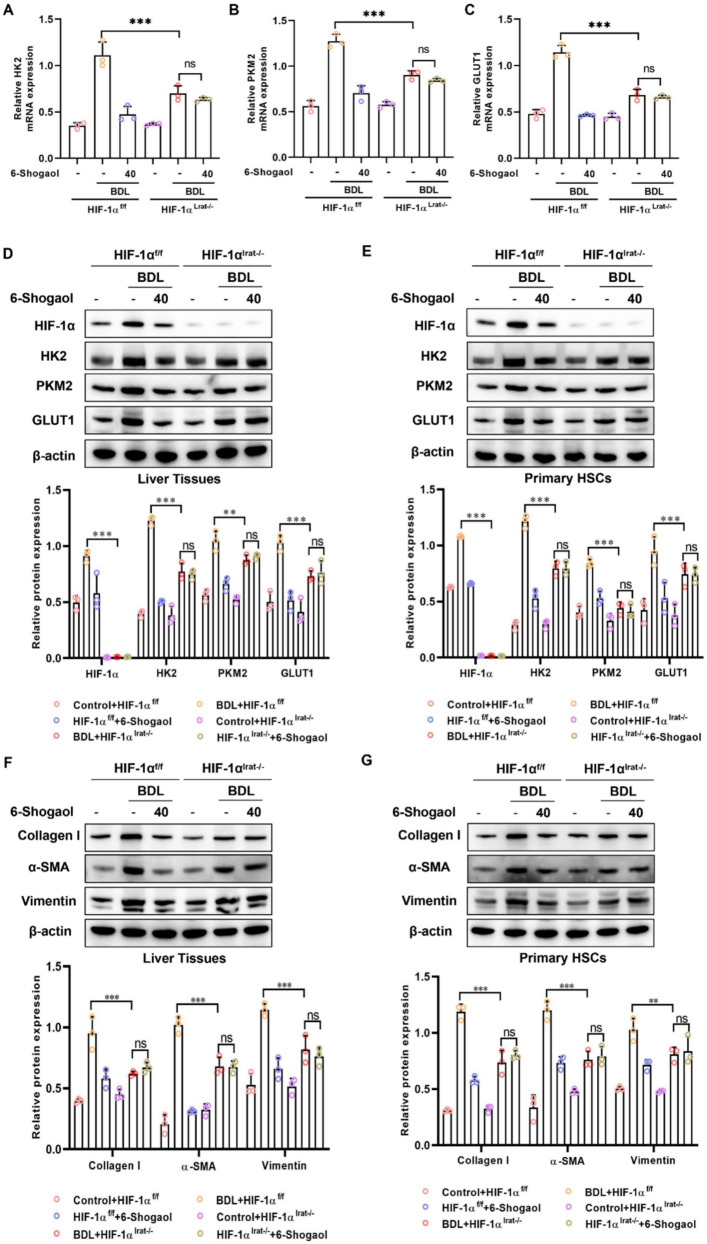
HIF-1α deletion attenuates the anti-glycolytic and anti-fibrotic effects of 6-Shogaol in BDL-induced liver fibrosis and in primary HSCs. **A**, **B**, **C** The mRNA expression of HK2, PKM2 and GLUT1 in mice liver fibrosis tissues. **D**, **E** The protein expression of HIF-1α, HK2, PKM2 and GLUT1 in liver tissues and primary HSCs. **F**, **G** The protein level of collagen I, α-SMA, vimentin in liver tissues and primary HSCs. *p < *0.05*, **p < 0.01, ***p < *0.01,* ns, not significant

Histopathological analysis (H&E staining, Masson staining, and Sirius red staining) demonstrated that fibrotic damage and collagen deposition were alleviated by 6-Shogaol in HIF-1α^f/f^ mice, whereas this protective effect was diminished in HIF-1α^lrat−/−^ mice (Fig. 8D and Fig. 9D). Similarly, serum biomarkers AST, ALT, and TBIL were notably elevated in HIF-1α^f/f^ mice after CCl₄- and BDL-treatments, indicating significant liver injury, whereas these markers were reduced in HIF-1α^lrat−/−^ mice (Fig. 8E–G and Fig. 9E–G). IHC staining revealed that 6-Shogaol downregulated the expression of glycolytic proteins (HK2, PKM2, and GLUT1), and fibrosis markers (collagen I and α-SMA) in liver tissues of HIF-1α^f/f^ mice, but not in HIF-1α^lrat−/−^ mice (Fig. 8H and Fig. 9H). In parallel, RT-qPCR results indicated that 6-Shogaol suppressed the mRNA levels of glycolysis-related genes (HK2, PKM2, and GLUT1) only in HIF-1α^f/f^ mice (Fig. 10A–C and Fig. 11A–C). Consistent with the above data, western blotting analysis confirmed that HIF-1α knockout reduced glycolytic proteins (HK2, PKM2, and GLUT1), and fibrosis markers (collagen I, α-SMA, and vimentin) protein levels, with 6-Shogaol treatment further potentiating this suppression in CCl₄- and BDL-treated HIF-1α^f/f^ mice (Fig. 10D and F; Fig. 11D and F). Primary HSCs were isolated from CCl₄- and BDL-treated HIF-1α^f/f^ and HIF-1α^lrat−/−^ mice to confirm the HSC-intrinsic role of HIF-1α. We found that 6-Shogaol greatly decreased the expression of glycolysis- and fibrosis-related proteins in primary HSCs from HIF-1α^f/f^ mice, whereas in HIF-1α-deficient primary HSCs, no such effect was observed (Fig. 10E and G; Fig. 11E and G). Collectively, these results suggested that HIF-1α deficiency effectively attenuated glycolytic metabolism and liver fibrosis in vivo, underscoring the therapeutic potential of targeting HIF-1α signaling pathways for liver fibrosis.

## Discussion

The pathological progression of liver fibrosis is characterized by chronic liver injury different etiologies as well as excessive accumulation of ECM. The continuous progression of fibrosis has been recognized as a core pathological process in cirrhosis and end-stage liver failure [44, 45]. Liver fibrosis can be categorized into two major types based on the mechanism of injury: hepatotoxic injury and cholestatic injury. Studies involving patients with liver fibrosis and cirrhosis of different etiologies, as well as experimental rodent models, have revealed the core molecular pathways associated with fibrosis. Specifically, the initial hepatocellular injury disrupts the epithelial/endothelial barrier, triggering an inflammatory cytokine cascade that ultimately promotes the differentiation of HSCs into collagen I-secreting myofibroblasts. This leads to the abnormal deposition of this protein-dominant ECM and the formation of fibrotic scar. Although certain drug candidates have entered clinical trials, no specific therapy is currently available for the treatment of liver fibrosis. In this context, active ingredients of traditional Chinese medicines with high efficiency and low toxicity are becoming an emerging strategic direction in the development of antifibrotic drugs owing to their multi-target modulation advantages.

Notably, 6-Shogaol, an active component extracted from dried ginger, has demonstrated considerable potential as an intervention for hepatic fibrosis owing to its multiple pharmacological activities, including antioxidant, anti-inflammatory, and anti-cancer properties. The potent antioxidant properties of 6-Shogaol have been reported to eliminate free radicals and protect neuron-like rat pheochromocytoma PC12 cells from oxidative stress injury [46]. In addition, 6-Shogaol exerted neuroprotective effects on dopaminergic neurons via anti-neuroinflammatory mechanisms in both in vitro and in vivo models of Parkinson’s disease [47]. Its anti-inflammatory properties have been observed in CCl_4_-induced liver fibrosis model, in which it effectively attenuated liver inflammatory responses [36]. Furthermore, Li et al. [48] reported that 6-Shogaol could inhibit the proliferation of human colon adenocarcinoma cells by inducing cell cycle arrest and promoting autophagy and apoptosis. Collectively, these studies suggested the great potential of 6-Shogaol for regulating HSCs activation. In this study, the antifibrotic effects and underlying mechanisms of 6-Shogaol were systematically evaluated using CCl_4_-induced liver fibrosis models and BDL-induced liver fibrosis models. We demonstrated that 6-Shogaol dose-dependently improved liver function indices such as serum ALT, AST, and TBIL in liver fibrosis mice, and its significant inhibitory effect on liver collagen deposition was confirmed by histopathological analysis (H&E, Masson and Sirus red). Similarly, molecular assays further revealed downregulation of mRNA and protein expression of liver fibrotic markers, including collagen I, α-SMA and vimentin, suggesting a direct inhibitory effect on HSCs activation. Notably, TGF-β1, a pro-fibrotic core factor, promoted ECM accumulation via HSCs activation [49, 50]. In the present study, 6-Shogaol markedly inhibited TGF-β1-induced phenotypic transformation of HSCs. These findings suggested that 6-Shogaol prevented ECM overproduction by targeting HSCs, supporting its potential as a therapeutic candidate for liver fibrosis. Although this study primarily examined the therapeutic efficacy and mechanism of 6-Shogaol, it is essential to briefly address the potential toxicity of 6-Shogaol and comprehensively evaluate its therapeutic profile. Previous studies demonstrated that 6-Shogaol, as a major bioactive compound present in ginger, is safe at therapeutic doses. They did not observe any significant adverse reactions or toxicity when administering 6-Shogaol at doses of 10 to 100 mg/kg to rodents [15, 46]. In our study, we did not observe any significant signs of toxicity or behavioral abnormalities in mice treated with 6-Shogaol at doses up to 40 mg/kg. Furthermore, 6-Shogaol concentrations up to 25 µM effectively suppressed HSCs activation without inducing notable cytotoxicity in LX-2 cells. Nevertheless, comprehensive toxicological studies need to be conducted in future preclinical developments, including detailed evaluations of organ-specific toxicity, the maximum tolerated dose, and potential long-term effects, to establish the safety window of 6-Shogaol and lay a solid foundation for its clinical translation in the treatment of chronic liver diseases.

Recent studies have highlighted the critical role of metabolic reprogramming in fibrosis [51–53], especially the link between enhanced glycolysis and HSCs activation [11, 54]. In the study, we observed that both the CCl_4_- and the BDL-induced liver fibrosis model, as well as TGF-β1-stimulated HSCs, exhibited a glycolytic phenotype marked by elevated glycolytic metabolites (lactic acid and pyruvic acid) and rate-limiting enzymes (HK2, PKM2, and GLUT1). Notably, 6-Shogaol suppressed HSCs activation via repressing glycolysis in a dose-dependent manner, and its antifibrotic efficacy was inversely correlated with glycolysis intensity. Therefore, it could be concluded that 6-Shogaol ameliorated liver fibrosis by inhibiting the glycolytic pathway and reducing HSCs glycolytic metabolism. Nevertheless, the exact mechanism by which 6-Shogaol regulates glycolysis to exert its antifibrotic effects needs to be further explored.

The role of HIF-1α in fibrotic diseases and glycolytic reprogramming has been increasingly recognized. HIF-1α enabled it to bind hypoxia-responsive elements in target gene promoters to regulate gene expression in response to oxygen availability, which plays a pivotal role in cellular adaptation to hypoxia, regulating processes such as angiogenesis, metabolism, and inflammation [55, 56]. A previous study confirmed the inhibition effect of 6-Shogaol on HIF-1α [57]. Herein, we identified HIF-1α as a common target of 6-Shogaol (the drug) and liver fibrosis (the disease) through network pharmacology analysis. Our RNA-seq data revealed that the expression of HIF-1α was significantly downregulated in fibrotic liver tissues after 6-Shogaol administration compared to that in untreated fibrotic tissues. Furthermore, increased HIF-1α expression accompanied fibrosis progression and was positively correlated with liver function and fibrosis markers in both *in vivo *and *in vitro* models. Thus, molecular mechanistic studies are essential to elucidate how abnormalities in glycolytic metabolism contribute to liver fibrosis and facilitate the translation of these findings into clinical applications. Next, our study highlights the crucial role of HIF-1α in regulating glycolysis during liver fibrosis, as demonstrated by both overexpression and knockdown experiments. The overexpression of HIF-1α in LX-2 cells, followed by TGF-β treatment, resulted in more severe fibrosis and elevated expression of glycolytic enzymes (HK2, PKM2, and GLUT1) compared to vector controls, consistent with increased levels of collagen I, α-SMA, and vimentin. In contrast, our findings demonstrated that HIF-1α knockdown inhibited the expression of glycolytic enzymes and fibrotic markers. However, 6-Shogaol failed to inhibit fibrosis and glycolysis in HIF-1α conditional knockout mice models, providing strong evidence for the target specificity of its action. These results suggested that 6-Shogaol reduced ECM production and the expression of key enzymes involved in glycolysis by downregulating HIF-1α, which is critical in the pathogenesis of liver fibrosis.

Given the relatively low cost and high safety profile of natural dietary compounds, 6-Shogaol holds significant potential for clinical translation as a therapeutic agent for liver fibrosis. Hence, this study primarily focused on the mechanism by which 6-Shogaol directly regulates the glycolytic reprogramming of HSCs through targeting HIF-1α, thereby inhibiting liver fibrosis. However, liver fibrosis is a complex pathological process that involved interactions between different cell types, such as hepatocytes and macrophages. Although our results demonstrated that 6-Shogaol could significantly improve the ECM deposition and metabolic status of the liver, this study had not yet been able to comprehensively analyze whether and how 6-Shogaol affects the metabolism of other cell types in the fibrosis microenvironment, such as by inhibiting the glycolysis of macrophages or protecting the metabolic function of hepatocytes. More importantly, this study has not yet explored the contribution of these cell-to-cell interactions (through metabolites, such as lactate or cytokines) to the antifibrotic effect of 6-Shogaol. This is a limitation of this study. Future research will utilize cell-specific knockout animal models, single-cell sequencing, and cell co-culture systems to further reveal the precise mechanism by which 6-Shogaol regulates the intercellular metabolic dialogue in the fibrotic microenvironment. Furthermore, based on network pharmacology analysis, we predicted multiple potential therapeutic targets of 6-Shogaol, including key signaling nodes such as TP53, AKT1, and TNF, suggesting that 6-Shogaol may possess anti-fibrotic properties through multi-target regulation. However, we focused on HIF-1α, a key transcription factor in metabolic regulation, for in-depth verification, due to the core role of glycolytic reprogramming in HSC activation and the implication of our preliminary omics data. We combined computational simulation, molecular binding experiments, cellular functional studies, and cell-specific gene knockout animal models to confirm the specific mechanism by which 6-Shogaol inhibits HSC activation by directly targeting HIF-1α and suppressing its mediated glycolytic program. The occurrence and development of fibrosis involve complex crosstalk between signaling networks. For example, the PI3K/AKT signaling pathway could upstream regulate the stability of HIF-1α, whereas the NF-κB signaling pathway could synergize with HIF-1α at inflammatory and metabolic levels. Therefore, whether 6-Shogaol exerts a synergistic effect by simultaneously regulating these interconnected pathway networks is an extremely important scientific question that remains to be elucidated. Future studies will explore the effects of 6-Shogaol on other predicted core targets and elucidate the crosstalk between the HIF-1α and key fibrosis-related pathways, such as PI3K/AKT signaling pathway and NF-κB signaling pathway, thereby more systematically revealing its multi-target pharmacological properties and synergistic antifibrotic mechanisms. To summarize, our study suggested that 6-Shogaol effectively ameliorated liver fibrosis by inhibiting HIF-1α and its downstream glycolytic signaling in mouse models and in cell cultures, offering a promising metabolic-based therapeutic approach.

## Conclusion

The present study demonstrated that 6-Shogaol greatly alleviated the pathological process of liver fibrosis by inhibiting HIF-1α expression and associated glycolytic reprogramming. These findings deepen our understanding of natural product-based antifibrotic mechanisms and provide a theoretical basis for the development of metabolism-targeted therapies for liver fibrosis.

## Supplementary Information

Supplementary File 1. Figure S1. (A) *In vitro*, CCK8 was used to explore the concentration of 6-Shogaol. (B) DARTS analysis identified the interaction between HIF-1α and 6-Shogaol in the LX-2 cells. (C) Immunofluorescence staining bespeak the colocalization of HIF-1α and α-SMA.

Supplementary File 2. Figure S2. (A) The contents of lactic acid and pyruvate acid in LX-2 cells transfected with PEX-3-PKM2. (B) The protein level of PKM2, α-SMA, vimentin in LX-2 cells transfected with PEX-3-PKM2. *p < 0.05, **p < 0.01, ***p < 0.01, ns, not significant.

## Data Availability

The datasets used and/or analyzed during the current study are available from the corresponding author on reasonable request.

## Abbreviations

CCl_4_: Carbon tetrachloride
BDL: Bile duct ligation
TGF-β1: Transforming growth factor-β1
HSCs: Hepatic stellate cells
DARTS: Reactivity target stability
ITC: And Isothermal titration calorimetry
OCR: Oxygen consumption rate
ECAR: Extracellular acidification rate
HIF-1α: Hypoxia-inducible factor-1 alpha
LDHA: Lactate dehydrogenase
GO: Gene Ontology
KEGG: Kyoto Encyclopedia of Genes and Genomes
ALT: Alanine aminotransferase
AST: Aspartate aminotransferase
TBIL: Levels of total bilirubin
GAPDH: Glyceraldehyde 3-phosphate dehydrogenase
cDNA: Complementary DNA
SDS-PAGE: Sulfate–polyacrylamide gel electrophoresis
DEG: Differential expression gene
siRNA: Small-interference RNA
OB: Oral bioavailability
Rg: Radius of gyration
RMSF: Root mean square fluctuation
