# Supplementary figures and images for: 6-Shogaol inhibits HSCs activation and liver fibrosis by regulating glycolytic reprogramming via targeting HIF-1α
Source: Chin Med. 2026 May 27;21:146. doi: 10.1186/s13020-026-01396-y (PMC13214082; doi:10.1186/s13020-026-01396-y)

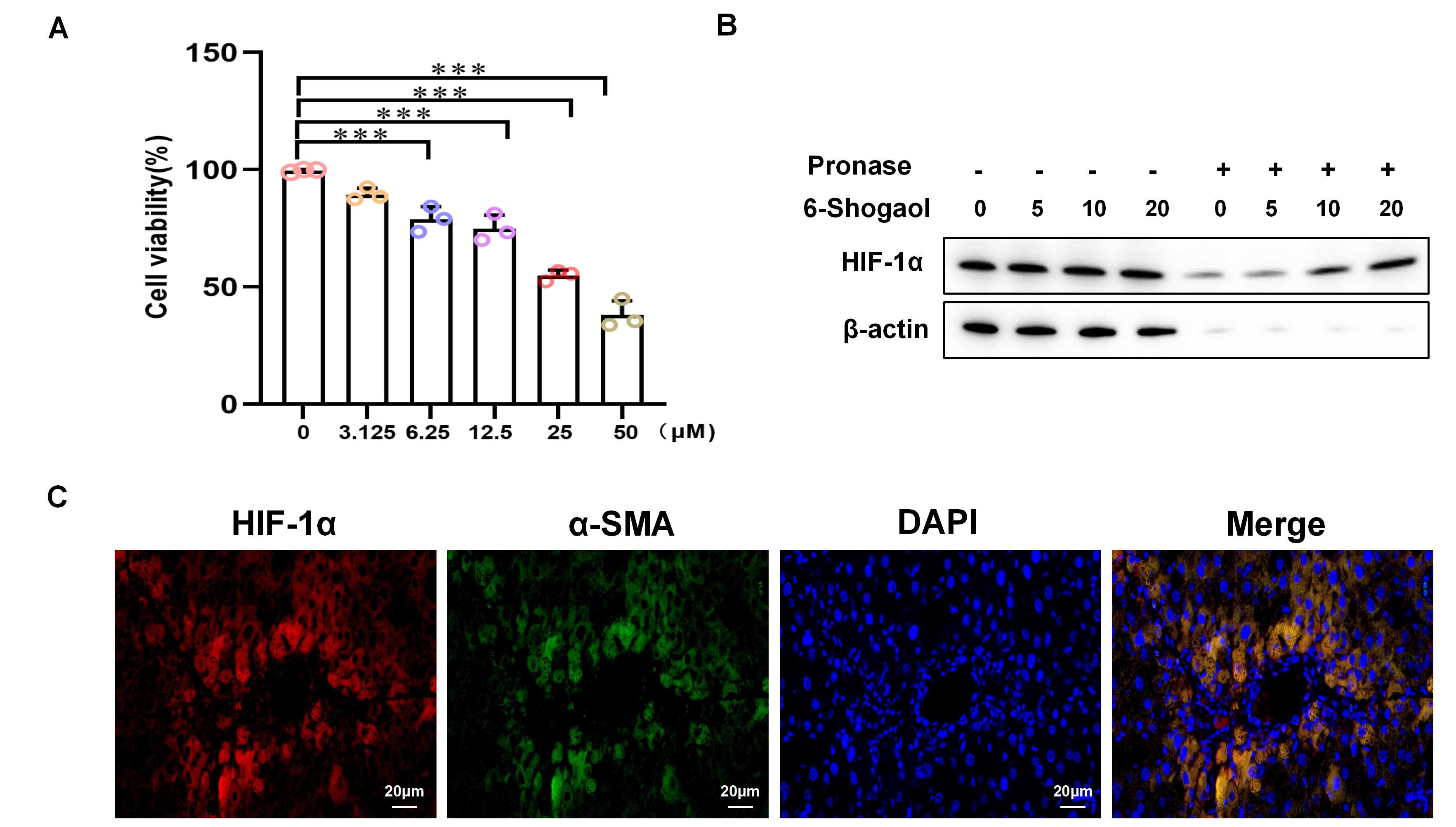

Supplement: Supplementary file 1 — Figure S1. (A) In vitro, CCK8 was used to explore the concentration of 6-Shogaol. (B) DARTS analysis identified the interaction between HIF-1α and 6-Shogaol in the LX-2 cells. (C) Immunofluorescence staining bespeak the colocalization of HIF-1α and α-SMA. [file 13020_2026_1396_MOESM1_ESM.jpg]
